# Exploring the floristic diversity of tropical Africa

**DOI:** 10.1186/s12915-017-0356-8

**Published:** 2017-03-07

**Authors:** Marc S. M. Sosef, Gilles Dauby, Anne Blach-Overgaard, Xander van der Burgt, Luís Catarino, Theo Damen, Vincent Deblauwe, Steven Dessein, John Dransfield, Vincent Droissart, Maria Cristina Duarte, Henry Engledow, Geoffrey Fadeur, Rui Figueira, Roy E. Gereau, Olivier J. Hardy, David J. Harris, Janneke de Heij, Steven Janssens, Yannick Klomberg, Alexandra C. Ley, Barbara A. Mackinder, Pierre Meerts, Jeike L. van de Poel, Bonaventure Sonké, Tariq Stévart, Piet Stoffelen, Jens-Christian Svenning, Pierre Sepulchre, Rainer Zaiss, Jan J. Wieringa, Thomas L. P. Couvreur

**Affiliations:** 1 Botanic Garden Meise, Nieuwelaan 38, BE-1860 Meise, Belgium; 2 DIADE, Université Montpellier, IRD, Montpellier, France; 3 Section for Ecoinformatics & Biodiversity, Department of Bioscience, Aarhus University, Ny Munkegade 114, DK-8000 Aarhus C, Denmark; 4 Royal Botanic Gardens, Kew, Richmond, Surrey, TW9 3AE UK; 5 Centre for Ecology, Evolution and Environmental Changes (CE3C), Faculty of Sciences, University of Lisbon, Campo Grande, 1749-016 Lisbon, Portugal; 6 Wageningen University, Biosystematics Group, Droevendaalsesteeg 1, 6708 PB Wageningen, The Netherlands; 7 Herbarium et Bibliothèque de Botanique Africaine, Université Libre de Bruxelles, Boulevard du Triomphe, B-1050 Bruxelles, Belgium; 8 Laboratoire de Botanique systématique et d’Écologie, Département des Sciences Biologiques, École Normale Supérieure, Université de Yaoundé I, Yaoundé, Cameroon; 9 Missouri Botanical Garden, Africa & Madagascar Department, P.O. Box 299, St. Louis, Missouri 63166-0299 USA; 10 CIBIO/InBio, Centro de Investigação em Biodiversidade e Recursos Genéticos, Universidade do Porto, Campus Agrário de Vairão, Vairão, Portugal; 11 CEABN/InBio, Centro de Ecologia Aplicada “Professor Baeta Neves”, Instituto Superior de Agronomia, Universidade de Lisboa, Tapada da Ajuda, 1349-017 Lisboa Portugal; 12 Laboratoire d’Évolution biologique et Écologie, Faculté des Sciences, Université Libre de Bruxelles, Brussels, Belgium; 13 Royal Botanic Garden Edinburgh, 20A Inverleith Row, Edinburgh, UK; 14 Naturalis Biodiversity Center, Darwinweg 2, 2333 CR Leiden, The Netherlands; 15 Picturae, De Droogmakerij 12, 1851LX Heiloo, The Netherlands; 16 Department of Ecology, Faculty of Science, Charles University, Vinicna 7, 128 44 Prague 2, Czech Republic; 17 Institut für Geobotanik und Botanischer Garten, Im Neuwerk 21, University Halle-Wittenberg, 06108 Halle (Saale), Germany; 18 Laboratoire d’Ecologie végétale et Biogéochimie, Université Libre de Bruxelles, Boulevard du Triomphe, B-1050 Bruxelles, Belgium; 19 Laboratoire des Sciences du Climat et de l’Environnement, LSCE/IPSL, CEA-CNRSUVSQ, Université Paris-Saclay, F-91191 Gif-sur-Yvette, France; 20 AMAP, CNRS, INRA, IRD, Université Montpellier, Montpellier, France; 21 CESAB/FRB, Domaine du Petit Arbois, Av. Louis Philibert, Aix-en-Provence, 13100 France; 22 Center for Tropical Research, Institute of the Environment and Sustainability, University of California, Los Angeles, Box 951496, Los Angeles, CA 90095 USA; 23 International Institute of Tropical Agriculture, BP 2008 (Messa), Yaounde, Cameroon

**Keywords:** Herbarium specimens, Digitization, Tropical forests, Botanical exploration, Plant growth form, Species richness, Floristic patterns

## Abstract

**Background:**

Understanding the patterns of biodiversity distribution and what influences them is a fundamental pre-requisite for effective conservation and sustainable utilisation of biodiversity. Such knowledge is increasingly urgent as biodiversity responds to the ongoing effects of global climate change. Nowhere is this more acute than in species-rich tropical Africa, where so little is known about plant diversity and its distribution. In this paper, we use RAINBIO – one of the largest mega-databases of tropical African vascular plant species distributions ever compiled – to address questions about plant and growth form diversity across tropical Africa.

**Results:**

The filtered RAINBIO dataset contains 609,776 georeferenced records representing 22,577 species. Growth form data are recorded for 97% of all species. Records are well distributed, but heterogeneous across the continent. Overall, tropical Africa remains poorly sampled. When using sampling units (SU) of 0.5°, just 21 reach appropriate collection density and sampling completeness, and the average number of records per species per SU is only 1.84. Species richness (observed and estimated) and endemism figures per country are provided. Benin, Cameroon, Gabon, Ivory Coast and Liberia appear as the botanically best-explored countries, but none are optimally explored. Forests in the region contain 15,387 vascular plant species, of which 3013 are trees, representing 5–7% of the estimated world’s tropical tree flora. The central African forests have the highest endemism rate across Africa, with approximately 30% of species being endemic.

**Conclusions:**

The botanical exploration of tropical Africa is far from complete, underlining the need for intensified inventories and digitization. We propose priority target areas for future sampling efforts, mainly focused on Tanzania, Atlantic Central Africa and West Africa. The observed number of tree species for African forests is smaller than those estimated from global tree data, suggesting that a significant number of species are yet to be discovered. Our data provide a solid basis for a more sustainable management and improved conservation of tropical Africa’s unique flora, and is important for achieving Objective 1 of the Global Strategy for Plant Conservation 2011–2020. In turn, RAINBIO provides a solid basis for a more sustainable management and improved conservation of tropical Africa’s unique flora.

**Electronic supplementary material:**

The online version of this article (doi:10.1186/s12915-017-0356-8) contains supplementary material, which is available to authorized users.

## Background

**Fig. 1:**
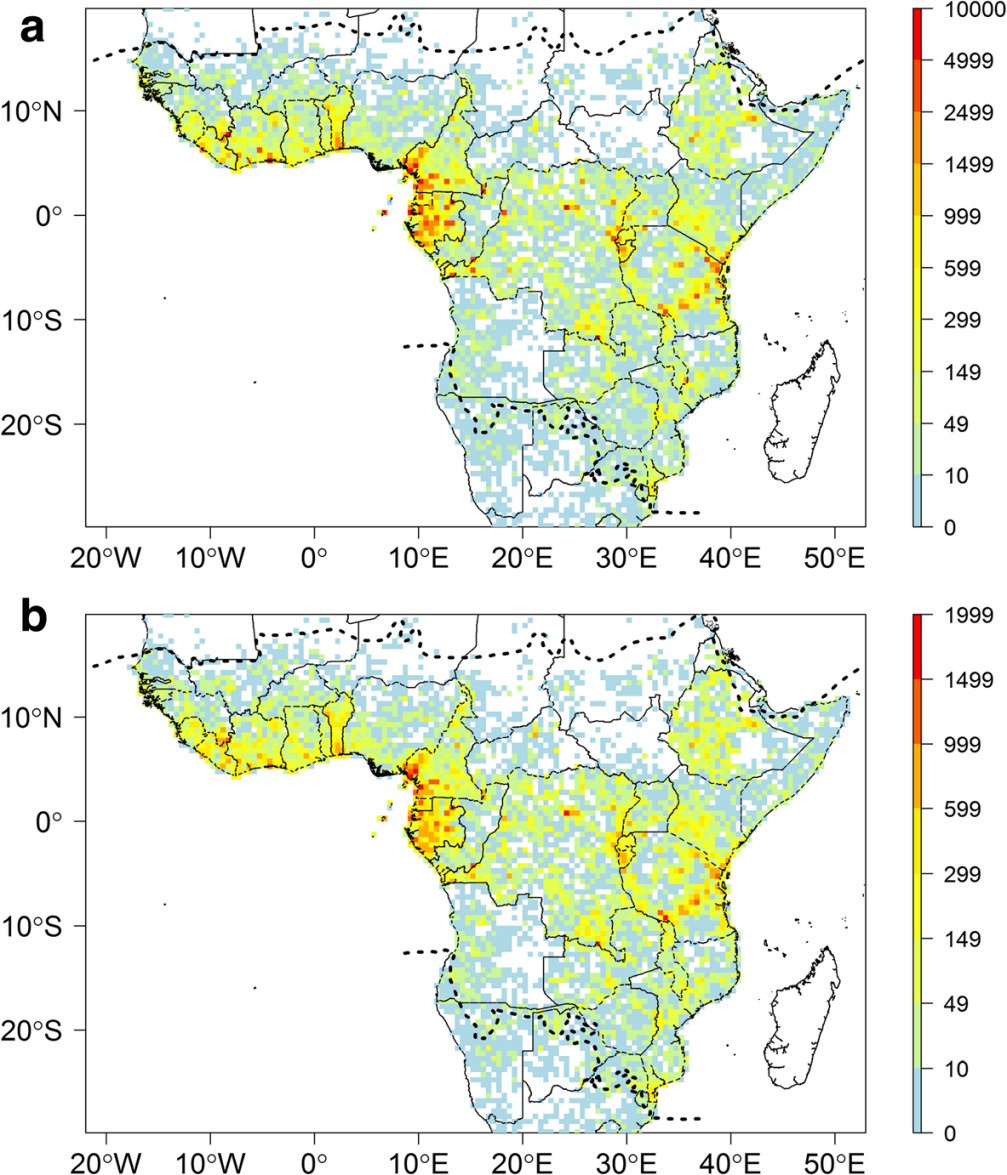
Distribution of botanical records across tropical Africa. Number of specimens (**a**) and observed species richness (**b**) per 0.5° sampling units. Dashed lines represent the limits for tropical Africa as defined in our study. Map based on georeferenced herbarium records, silica gel samples and plot data

Documenting the distribution of biodiversity is the first and most fundamental step for effective conservation and sustainable utilisation of natural resources for the future [1]. Tropical Africa [2] (Fig. 1) is home to some of the most important species-rich biodiversity regions in the world [3]. From the second largest extent of continuous rain forest in the world, the Congo basin, to the Namib dessert, tropical Africa is a land of strong biodiversity contrasts [4]. Yet, it has already lost large amounts of its ‘wilderness areas’ [5], i.e. areas where ecological and evolutionary processes are little affected by human disturbance. In addition, future climate change is expected to have important negative effects on sub-Saharan ecosystems, with an estimated 90% of species loosing part or most of their areas of suitable climate by 2085 [6]. These results call for immediate international policies to be put in place [5, 7, 8]; however, they will be hard to achieve without a better understanding of plant diversity and its distribution.

Herbaria around the world are the keepers of such essential data for plants [7, 9, 10]. Together, they curate a vast databank of about 350,000,000 physical dried plant specimens [11]. Any conserved specimen provides unique proof of where and when a particular species was present. With ongoing and intensified efforts of natural history institutions to digitize their scientific heritage, the possibility of providing large amounts of data to a much wider audience has rapidly expanded over the last few years [12–16] (see [17] for an overview of the number of scanned herbarium specimens in the world’s largest virtual herbaria).

Relying on such data, several large-scale initiatives have been undertaken focusing on, for example, the tree flora of the Amazon basin [18–20], enabling access to more detailed estimates on plant diversity and distribution in this species-rich region. To date, however, our knowledge of tropical plant distribution remains limited [21–23] and tropical Africa is no exception to the rule [24, 25]. Indeed, total species estimates for different countries, regions and biomes are lacking or are approximate estimates at best. For example, White [26] in his much-cited *The Vegetation of Africa* provided broad estimates of plant species diversity for each of his recognised ‘phytochoria’, numbers that are still used today. However, these values were not based on any formal analyses of biodiversity data, but rather on expert opinion. In addition, there have been numerous efforts to estimate the total number of tree species across the tropics, either in general for the tropics [27] or for specific regions such as the Amazon basin [20]. Few estimates of total tree diversity for Africa have been advanced. Using extrapolations from global plot data, Slik et al. [27] estimated a minimum number of 4626–5984 tree species for Africa (including Madagascar). In general, tropical Africa is deemed to be botanically less diverse than regions at similar latitudes in South America and South East Asia [27–29]. Numerous hypotheses exist which try to explain this ‘odd man out’ pattern (reviewed in [28]) but no clear conclusive explanation has yet been provided.

During the last 15 years, efforts to database tropical African plants have been undertaken, but generally with the purpose of analysing large scale phytogeographical patterns across Africa [30–32]. Thus, these datasets are characterised by an overrepresentation of well-known and comparatively widespread species. More recently, Stropp et al. [25] extracted and analysed all flowering plant data openly available via the Global Biodiversity Information Facility (GBIF, http://www.gbif.org) for Africa (including Madagascar). They concluded that the quality and completeness of species-occurrence data of flowering plant species available through Biodiversity Information Systems such as GBIF is low. In addition, over half of the records they retrieved did not relate to tropical Africa but to South Africa and Madagascar. Thus, to date, large uncertainties remain about plant species diversity and distribution across tropical Africa. The digitization and georeferencing of major African-specialised herbarium collections have substantially progressed these past years [16, 17]. When these independent efforts are compiled and concatenated into a single quality-checked comprehensive mega-database, they will provide new and detailed insights into tropical African plant diversity and distribution as never achieved before [33].

The goal of this paper is to document the main floristic patterns and information gaps for tropical Africa. For this, we use the largest and highest quality mega-database of vascular plant species distributions ever compiled for tropical Africa (RAINBIO, [33]). First, we explore the spatial distribution of records and species diversity statistics at regional and country levels and ask the following questions: how is plant species diversity distributed across tropical Africa? How many plant species are there in tropical African forests? How is this diversity partitioned in terms of growth form? Which regions have the highest floristic turnover rates? Second, we analyse the level of botanical exploration across tropical Africa and ask the following questions: have tropical African countries been adequately explored? What regions are well sampled and which are not? Can we identify areas where future sampling would be the most efficient? Finally, we conclude with a series of recommendations in order to improve our knowledge of the floristic diversity of tropical Africa.

## Methods

### Data and study area

All analyses were performed using different subsets (i.e. with or without silica gel information, see below) of the RAINBIO database [33]. In short, RAINBIO is a compilation of:

- publicly available datasets mainly from international herbaria;
- personal ones such as datasets on palms, legumes, orchids, Rubiaceae, Marantaceae, flora of Gabon, flora of the Dzanga-Sangha region (Central African Republic);
- tree plot inventory data from Gabon;
- georeferenced silica-dried samples for tree species from Central Africa.

The data were checked automatically and manually via experts for quality in several ways, including geographic and taxonomic standardisation, merging of duplicate records (originating from different datasets), and exclusion of cultivated/non-native plants. A full description of RAINBIO, how it was compiled and how the data were checked and verified can be found in Dauby et al. [33]. The RAINBIO dataset contains important additions and a significant increase in data quality compared to the data available through the GBIF (see, [25] for biodiversity analyses of data only available via GBIF), and has a strong focus on tropical Africa [33].

Our study area is tropical Africa, which Klopper et al. [2] broadly defined as sub-Saharan Africa and excludes southern Africa and Madagascar (Fig. 1, roughly between 16°N and 20°S). For delimitating this study area, we relied on the ecoregions defined by Olson et al. [34] and used the eco-region ‘south Saharan steppe and woodlands’ as the northern limit and ecoregions ‘Namibian savanna woodlands’, ‘Kalahari xeric savanna’, ‘Kalahari Acacia-Baikiaea woodland’, ‘Highveld grasslands’ and ‘Drakensberg montane grassland’ as the southern limits. We filtered the RAINBIO dataset so species having all of their occurrence records outside this region were excluded.

### Sampling unit size

In our study, we used two different sampling unit (SU) sizes. First, we simply used a ‘fixed-size’ SU of 0.5° (ca. 55 × 55 km at the equator). For certain analyses, however, we used an ‘adaptive resolution’ SU method [35]. This approach adapts the size of the SU as a function of a user-defined threshold of minimum occurrence records (see [35] for details). This method allows one to consider smaller SUs where record density is high, while increasing SUs size where records are sparsely distributed. The advantage is that record numbers remain broadly constant across SUs. In order to create an adaptive SU grid we uploaded the filtered RAINBIO database to the Infomap Bioregions [35] website (http://bioregions.mapequation.org). We used the following parameters: maximum cell capacity = 1000; minimum cell capacity = 500; maximum cell size = 8°; minimum cell size = 0.5°. The SU grid was then downloaded as a shapefile and processed in the R environment for excluding unsuitable areas (ocean and lakes) in each SU.

### Species diversity patterns

Species richness was estimated at two different levels: (1) for each fixed-size SU of 0.5° containing at least 100 records, and (2) at country level. Only herbarium specimens were included for this estimation. Plot data and silica gel-dried DNA samples were excluded because of their stronger focus on particular taxa, which are often over-sampled and would introduce a bias. In each case, an observed and estimated total species number was calculated. For the observed species number per country we used georeferenced and non-georeferenced specimens. As species richness estimation is strongly affected by sampling efforts, we computed two complementary richness estimators. First, we calculated a non-parametric species richness estimator [36], known as the *Chao1* [37], *Ŝ*, defined as:

$$ \widehat{S}={S}_{obs}+\frac{a_1^2}{2{a}_2}\frac{N-1}{N} $$

Where *a*_*1*_ is the number of species represented by one specimen (singleton), *a*_*2*_ is the number of species represented by two specimens (doubleton), *S*_*obs*_ is the observed number of species and *N* is the total number of specimens. Second, we estimated species richness using a subsampling procedure. This approach has proven to be robust when dealing with incomplete and heterogeneous sampling [38–40]. We used the Nielsen’s estimator *N*_*e*_ [41] of effective number of species [39], which corresponds to a nearly unbiased estimator of the popular Gini-Simpson diversity index [37] converted into effective number of species [38].

This estimator is defined as [41]:

$$ {N}_e=\frac{{\left( N-1\right)}^2}{3- N+\left( N+1\right)\left( N-2\right){\displaystyle {\sum}_s^i}{p_i}^2} $$

Where *N* is the total number of specimens, *p*_*i*_ is the frequency of the *i* ^th^ species and *S* is the observed number of species. This estimator does not estimate total species richness, but expresses the diversity in terms of ‘effective number of species’. In addition to its good statistical properties [38], this metric has the advantage of satisfying the ‘replication principle’ [37]. This basically means that the ratio of diversity values reflects ‘true’ differences in diversity just as actual species richness would do [42].

### Floristic turnover rates

- We assessed the level at which floristic composition changes in space by computing a ‘neighbourhood’ species turnover rate applying a three-step procedure (Additional file 1). For this parameter, we used the adaptive resolution SU grid as defined above.
- **Step 1.** For a focal SU, a convex hull was drawn around all specimen occurrences it contained. A given buffer of distance *μ* was added to the hull convex. All other SUs with at least 500 records included in this buffered area were selected for comparison.
- **Step 2.** The pairwise floristic similarity was computed as 1–*β*_*sim*_ between all selected SUs. The turnover index *β*_*sim*_ was defined as [43]: ${\beta}_{sim}\frac{min\left( b, c\right)}{a+ min\left( b, c\right)}$ where *b* and *c* are the number of species restricted to the first and second SU, respectively, while *a* is the number of species shared by the two cells.
- **Step 3.** The geographical distance among all pairs of selected cells was computed based on centroids of occurrences within each SU.

The decay of the floristic similarity with geographical distance was then approximated by a linear model. From this model, we extract the halving distance which was the geographical distance at which the floristic similarity was reduced to 50% [44]. Hence, low halving distances indicated high floristic turnover per distance unit. By changing the distance which determines the neighbourhood SUs that are taken into account (*μ*), one can investigate different scales of floristic turnover (small *μ* = fine-scale; large *μ* = large-scale) (see Additional file 1 for illustration of the method). Here, we investigate two values of *μ*: *μ* = 1°, representing a meso-scale floristic turnover, and *μ* = 2°, representing a large scale floristic turnover.

### Sampling completeness

Two estimates of sampling completeness were computed. First, the relative exploration was estimated as the percentage of species already discovered: observed species divided by the estimate of total number of species by Chao1. Second, the sampling coverage estimator of [45] was calculated as:

$$ {\widehat{C}}_N=1-\frac{a_1}{N}\left\lfloor \frac{\left( N-1\right){a}_1}{\left( N-1\right){a}_1+2{a}_2}\right\rfloor $$

A sampling coverage of 1 indicates that all species have been collected twice or more, while for a value of 0 all species are known by a single record only.

We identified the ‘best-explored’ countries, namely countries with a relative exploration value above 0.85 and sampling coverage higher than 0.95. However, this does not imply they were botanically well known, but rather, compared to others, they had the best data available to date. Figures for countries where the number of specimens was lower than the estimated number of species were deemed unreliable and thus not included in the results; obviously, these countries were poorly explored (or our data for them was largely incomplete).

Using adaptive resolution SUs we identified three major categories of SUs depending on their level of sampling and coverage. First, we defined ‘well-sampled units’ (WSU) representing SUs with a minimum collection density of 100 records for 100 km^2^ [46] and a sampling coverage estimator equal to or higher than 0.5. Second, we defined ‘top sampled units’ (TSU) as the top 25% of SUs with the highest density records and sampling coverage estimator equal to or higher than 0.5 and excluding WSUs. Third, we proposed a number of ‘priority sampling units’ (PSU) defined as a SU where meso-scale species turnover (see Floristic turnover rates section) was estimated to be high (the top 20% of SUs with lower halving distances), but where sampling completeness was low (smaller than 0.5). Thus, PSUs translate the idea that sampling should focus on poorly known areas with sharp floristic gradients. For example, SUs with low coverage but located in low turnover regions will be important to explore but might not lead to the addition of many new species for the region, if a nearby SU has already been well sampled. This approach should be viewed as complementary to other methods that have identified data-deficient areas in Africa [24, 25].

Finally, we estimated the level of sampling completeness across all tropical African species. For that, we assessed the number of specimens for each species as a function of the number of fixed-sized SUs occupied by that species. A scatter plot was then produced where each point represented a species, and the slope represented the average number of specimens per grid cell for all species. In order to have an idea of the historical evolution of this value through time, we generated the same plots but at 13 different time slices in the past, going back every 10 years until 1900.

All computations were done within the R statistical software (R Core Team 2015) using the *vegan* package [47] for the Chao1 estimator, *entropart* [48] for the sampling completeness estimator, *betapart* [49] for the *β*_*sim*_ computation and an R function built by G. Dauby to estimate the Nielsen parameter (Additional file 2).

### Estimation of species distributions

The distribution surface, or range size, of each species was estimated by calculating the surface within the convex hull formed by all specimen localities of that species. Large salt water bodies were excluded from the surface measurements. Species with a single georeferenced specimen were assigned an arbitrary range size of 1 km^2^. For species known from only two georeferenced specimens, the range size was estimated arbitrarily by multiplying the distance between the two localities by 0.1.

### Growth form diversity

Most species (91%) recorded in RAINBIO were able to be categorised into nine different growth form types (tree, shrub, herb, liana, vine, aquatic herb, epiphyte, mycoheterotroph and parasitic) [33]. For detailed information on how this was achieved, please see [33]. In short, main categories were automatically assigned through an ad hoc custom R script by extracting keywords for each habit from available herbarium specimen label note information. A total of 4751 species names (22% of the total filtered dataset, see below) were manually checked by experts in order to confirm automatic assignments (when two or more habits were suggested, 2823 species) and to fill in missing assignments (1928 species). Besides these specific cases, a large majority of habit assignments were confirmed (but no proportion can be provided). We considered species to be woody if they were classified as trees, shrubs or lianas, and as herbaceous when they were classified as herbs, vines, aquatics or mycoheterotrophs (parasitic species were not included because they are a mixture of both woody and herbaceous species). For each adaptive resolution SU we generated the total number of species, and the ratio of the number of species in each of five growth forms: (1) herbs, vines and mycoheterotrophs, (2) shrubs, (3) liana, (4) trees, and (5) epiphytes.

### African tropical forest diversity

In order to provide species diversity values for African tropical forests, we used the map of Mayaux et al. [50] depicting land cover types across Africa and Madagascar for the year 2000. The map consists of 27 different land cover layers. We selected six of them that consisted mostly, or originally, of tropical forests, namely evergreen forests, degraded evergreen forests, submontane forests, montane forests, swamp forests and mosaic forest-croplands. The original resolution of the map is 1 km^2^. We aggregated the selected land cover at a resolution of 0.1° (ca. 11 km^2^) because our georeferenced records are generally not that precise. The resulting forest area layer was then arbitrarily divided into a West African block (west to the Dahomey Gap), a central African block (east of the Dahomey Gap, west of the East African rift), and an eastern African block (east of the East African rift) (Additional file 3). Using these subunits we estimated the number of records, species richness, and number of sub-endemics (considered as a species with 90% or more of its records located within the forest layer) in total and for five different growth forms defined above for African forests in general and for each forest bloc (west, central and east).

## Results

### Spatial distribution of data

The original unfiltered RAINBIO database comprises distribution data for 25,356 species of vascular plants, 3158 genera and 273 families from a total of 614,022 records. The filtered dataset for tropical Africa that was used for our analyses had a total of 609,776 specimens representing 22,577 species. This represents the largest and highest quality dataset ever compiled for tropical African plants. The records are well distributed but heterogeneous across the continent (Fig. 1a). The highest collecting efforts are concentrated in West Africa (Liberia to Benin), south-western Cameroon, Equatorial Guinea, Gabon, the region covering the eastern part (Kivu) of Democratic Republic of the Congo (DRC) plus Rwanda and Burundi, and the Eastern Arc Mountain range in Kenya and Tanzania (Fig. 1a). Although to a lesser extent, records are also well represented in western and south-eastern DRC and Ethiopia. In contrast, there are comparatively few records in most of the Sahelian region (Senegal to South Sudan), the Ogaden (south-eastern Ethiopia), the Central African Republic, Nigeria, the Republic of Congo and Angola.

**Fig. 2:**
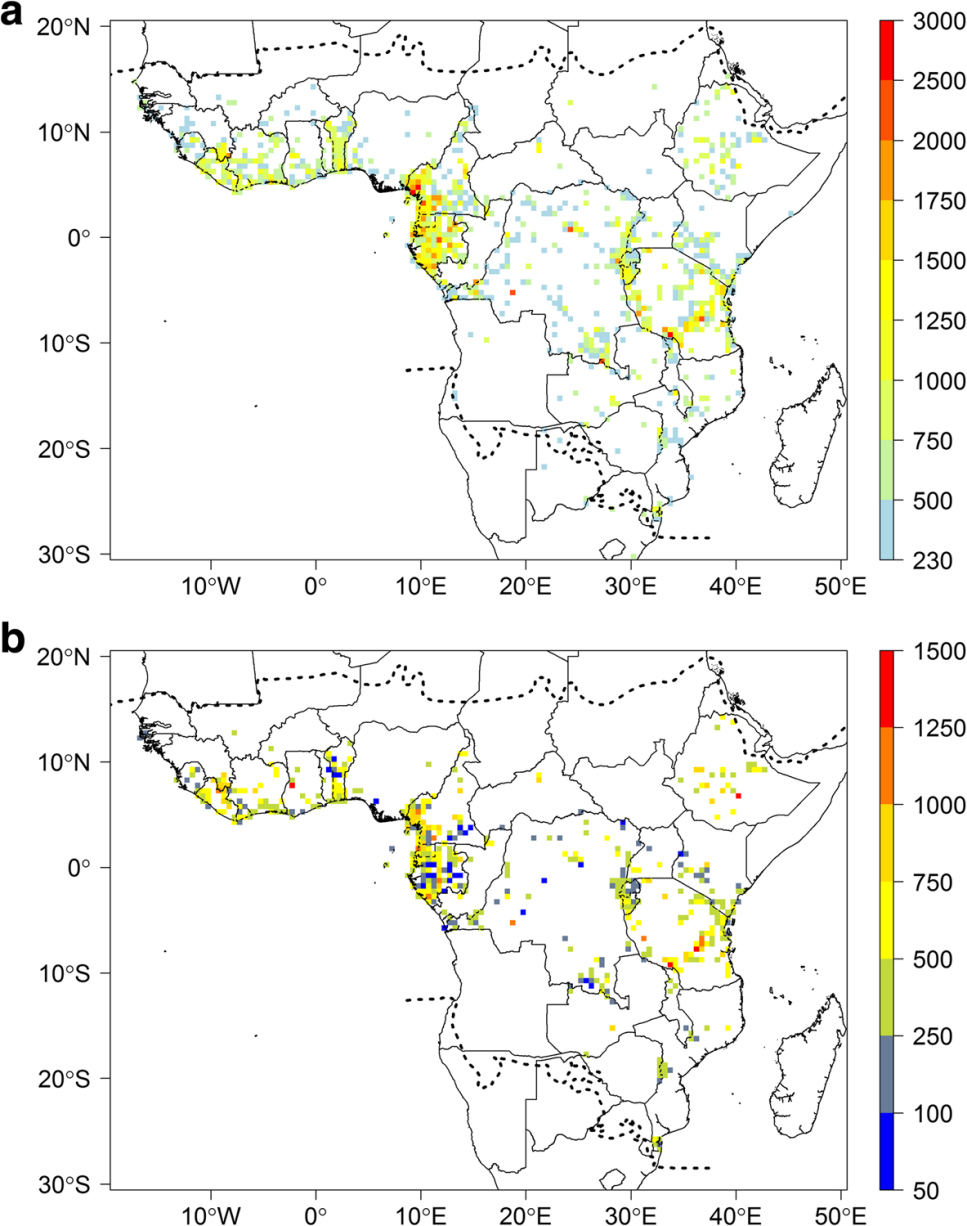
Estimated botanical diversity of tropical Africa. **a** Estimated species diversity based on the Chao1 estimator for each 0.5° sampling units (SUs) with more than 100 records. **b** Effective number of species estimated using the Nielsen statistic per 0.5° SUs with more than 100 records. Dashed lines represent the limits for tropical Africa as defined in our study. This map is based on georeferenced herbarium records, silica gel samples and plot data

Our data indicates that just 21 SUs of 0.5° contain more than 3000 records or 100 records per 100 km^2^ (Additional file 4). The observed species richness highlights several regions (Fig. 1b), with the following areas standing out: the Nimba Mountains in north Liberia, the Cameroonian Volcanic Line in western Cameroon, the regions around Kribi, Bipindi and Yaoundé in Cameroon, Rio Muni (continental part of Equatorial Guinea), several areas in Gabon such as the surroundings of Libreville and Makokou, around Yangambi in DRC, the Eastern Arc Mountain range in Tanzania, Abidjan in Ivory Coast and the south of Benin (Fig. 1b). The observed species richness is highly correlated with specimen density (Pearson correlation *R* = 0.91; compare Fig. 1a). Estimated total species richness for each SU based on the Chao1 (Fig. 2a) is highly correlated to both the number of records per SU (Fig. 1a; Pearson correlation *R* = 0.85) and the observed species richness (Fig. 1b, Pearson correlation *R* = 0.91). In contrast, the Nielsen diversity estimator computed for each 0.5° SU (Fig. 2b) is much less correlated with specimen density (Pearson correlation *R* = 0.28) than the Chao1 estimator.

A total of 3438 species have been collected just once and 8026 collected less than 5 times, while 3172 species have been collected more than 50 times (Additional file 5a). A similar picture is found when looking at the number of 0.5° SUs occupied per species (Additional file 5b). Over 4294 species are only recorded in a single SU (narrow endemics), while 1607 species occur in 50 SUs or more (widespread). The range size distribution of all species (Additional file 5c) shows a relatively low number of exceptionally widespread species (i.e. more than 16,777,216 km^2^). Species with an ‘intermediate’ range size (i.e. between 8192 and 16,777,216 km^2^) are most common, while species with small distribution areas are again less common. However, because of the quadratic scale used on the x-axis (to show more detail in the lower categories), the picture actually shows an ever increasing number of species along the x-axis.

**Fig. 3:**
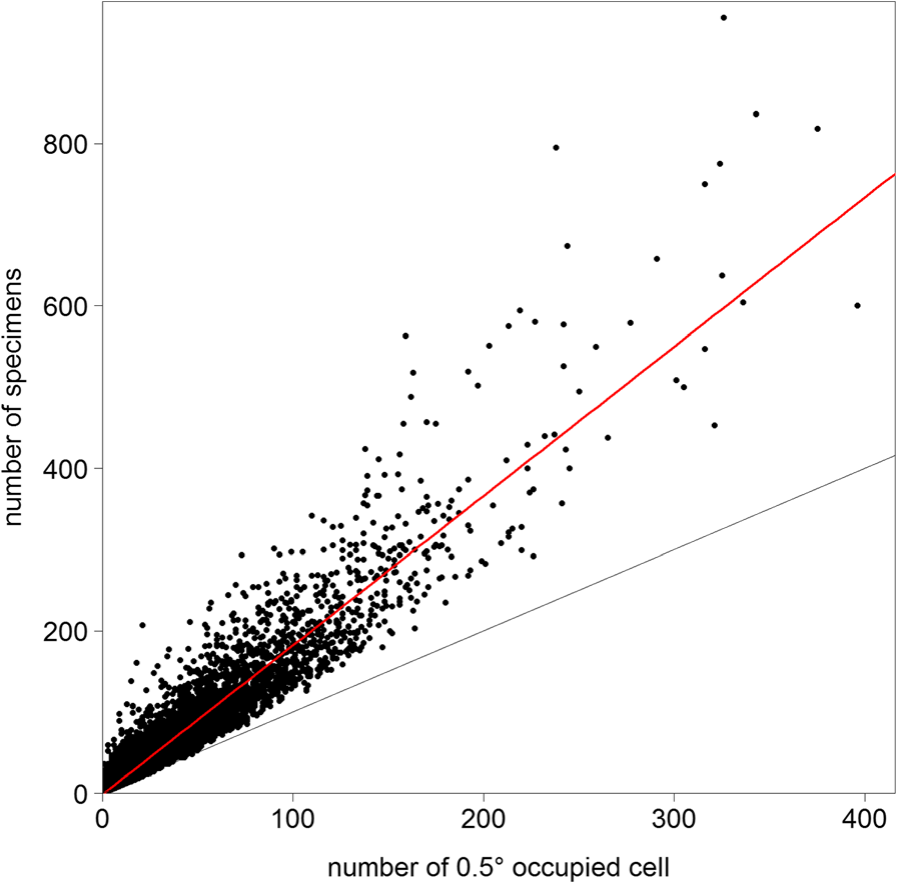
Spatial distribution of specimens per species. Each dot represents a species with its number of specimens against the number of 0.5° sampling units (SUs) it occupies. The slope of the red line (linear regression) indicates the average number of specimens per SU for all species. The grey line (slope equal to one) indicates when the number of specimens equals the number of occupied SUs

The average number of records collected per species per SU is 1.84, calculated as the slope of the linear fit of the numbers of specimens for each species as a function of the number of SUs it occupies (Fig. 3). Species that cluster on the lower left of the graph are limited in distribution while those to the right are comparatively widespread. Species that fall above the red line are collected more often than average while those under the red line are relatively under-collected. Rare species (left end of the plot) that fall above the red line are either locally common (clustered) or have been well-collected (e.g. by collectors focussing on specific groups), while those below the red line are comparatively scattered. Widespread species (right end of the plot) falling above the red line are comparatively abundant (widespread common) or have a positive collecting bias, those below the red line occur as scattered individuals (widespread rare) or are otherwise inconspicuous (minute or seasonal plants, like small saprophytes) or more difficult to collect (palms, large trees, aquatic plants or plants only flowering in the canopy) or have a negative collecting bias (e.g. weeds).

### Temporal distribution of collections

**Fig. 4:**
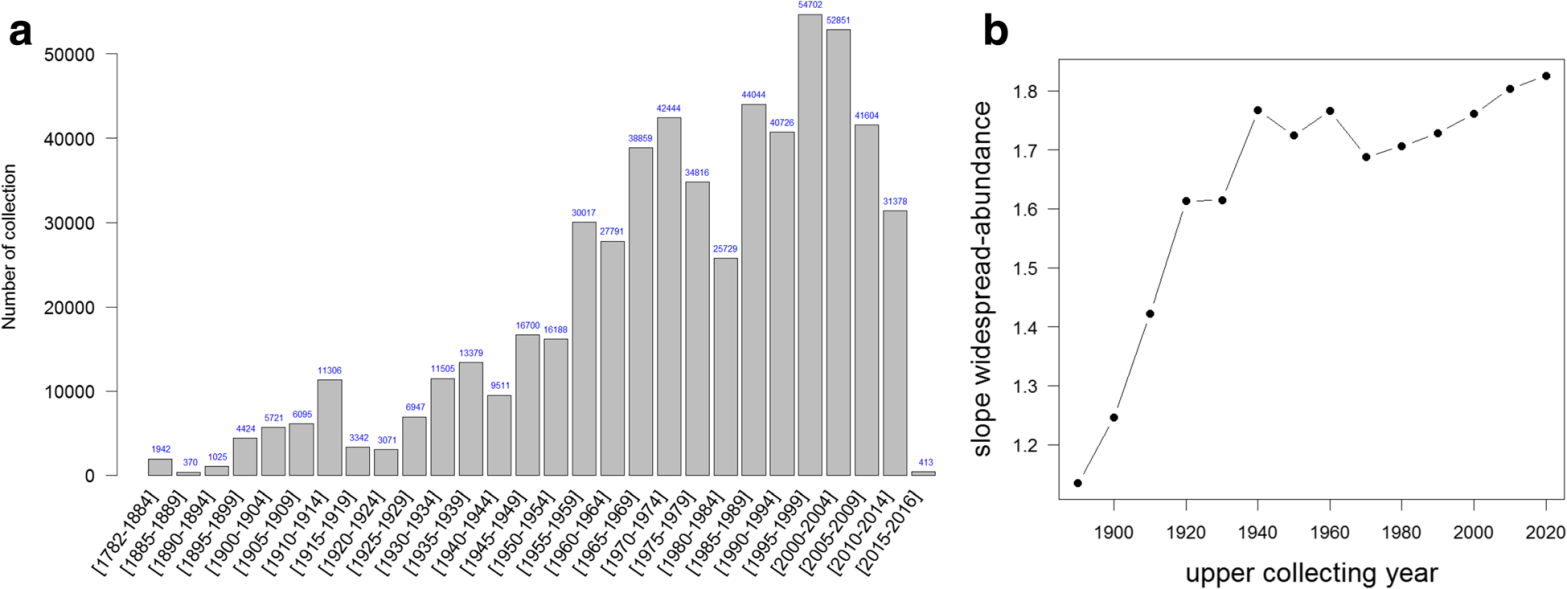
Temporal distribution of collecting efforts. **a** Number of herbarium records for tropical Africa per 5-year slices from 1782 to 2015. **b** Temporal evolution of the average number of specimens per SU for all species. Plots based on georeferenced herbarium records only

Collecting dates ranged from 1782 to 2015. Collecting intensity across tropical Africa through time has generally increased, though with lower collecting efforts in the early 1980s, to 2005, after which there has been a significant decline to the present (Fig. 4a). Collecting intensity per country shows different histories, with different periods of intense and low collecting efforts (Additional file 6). For example, DRC knew its highest period of botanical collections from the 1930s to the 1960s (Additional file 6). In contrast, Benin has experienced intense collecting over the last 20 years (Additional file 6). Other countries, such as Cameroon, have known a sustained and important collecting intensity since the 1960s up until 2010.

By plotting the average number of records collected per species per SU in function of 10-year time slices over 130 years, the slope is increasing, except for two periods (1941–1950 and 1961–1970) where the slope decreased (Fig. 4b). This is corroborated by a steady decrease of newly explored SUs since 1980 (Additional file 7).

**Fig. 5:**
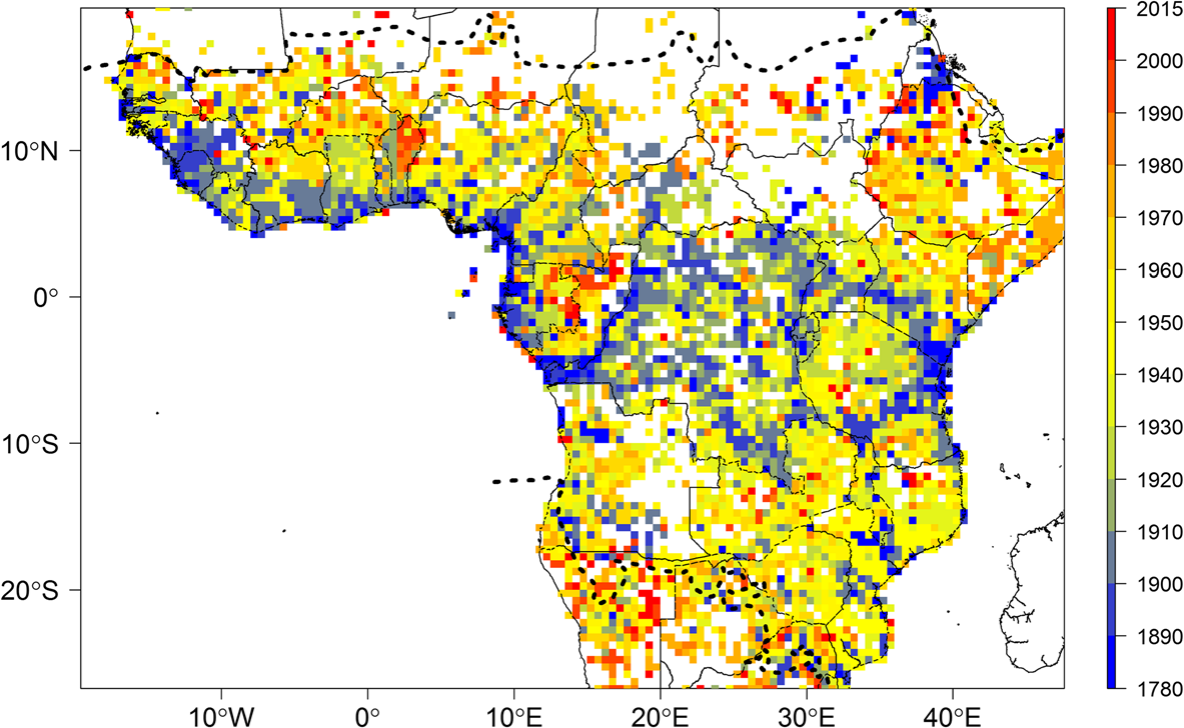
Time lapse of botanical collecting history across tropical Africa. The map represents the date of the first botanical collection made within each 0.5° sampling unit. Dashed lines represent the limits for tropical Africa as defined in our study. Map based on georeferenced herbarium records, silica gel samples and plot data. An animated gif version of this map is available at: http://rainbio.cesab.org

In order to have a ‘time lapse’ view of the collecting history throughout tropical Africa, we plotted the year of the oldest collection per SU from 1780 up to 2015 (Fig. 7, see Additional file 8). The first regions to be prospected (shades of blue in Fig. 5) are along the coast lines of East Africa (Kenya and Tanzania), central Africa (Cameroon, Equatorial Guinea, Gabon and Nigeria) and West Africa (Benin, Ghana, Guinea, Ivory Coast and Sierra Leone). Main rivers, such as the Congo River, were also explored during that time as well as mountain regions such as the Eastern Arcs in Tanzania, the Cameroonian Volcanic Range in Cameroon and the Crystal mountains in Gabon. The map also reveals areas that have been prospected only recently (shades of red in Fig. 5) such as the north of Benin, the Republic of Congo and the Haut-Ogooué region in Gabon. Some large areas that are still to be botanically explored (white in Fig. 5) are present along the southern margin of the Sahara, in eastern Chad, Sudan and South Sudan, northern and eastern Angola, and parts of the Ogaden desert in Ethiopia.

### Tropical African turnover rates

**Fig. 6:**
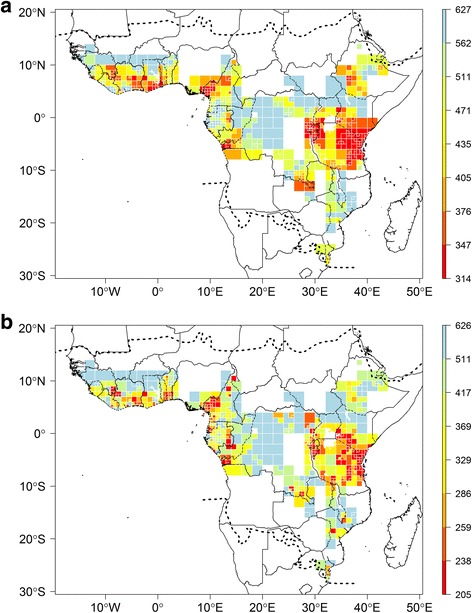
Floristic turnover rates across tropical Africa. Values based on adaptive resolution sampling unit (explanation see text). Pairwise floristic similarity is computed as 1–β_sim_ turnover index using two values of *μ* (see section in Methods and Additional file 1). **a** Meso-scale floristic turnover rate with *μ* = 1°; **b** Large-scale turnover rate with *μ* = 2°. Dashed lines represent the limits for tropical Africa as defined in our study. Maps based on georeferenced herbarium records, silica gel samples and plot data

High large-scale turnover rates (low halving distance values, Fig. 6a) are present in areas with high topographical heterogeneity, for example, along the Cameroonian Volcanic Range, the Albertine Rift or around the Nimba Mountains area, and forest-savannas transitional areas. The meso-scale turnover map (Fig. 6b) shows broadly similar areas, but also some striking differences. Some additional heterogeneous areas are identified, such as the regions around Libreville in Gabon and around Kribi and Bipindi in Cameroon, while others become homogeneous at the large scale.

### Botanical exploration intensity

The observed number of species and level of exploration of a selection of countries are provided in Table 1. The estimated botanically best-explored countries (Table 1, relative exploration index ≥ 0.85) are Cameroon, Benin, Gabon, Liberia and Ivory Coast. When looking at the sampling completeness, DRC and Tanzania can be added to this list (Table 1). The botanically least explored countries (relative exploration index ≤ 0.65) are Angola, Somalia, Botswana, Republic of Congo, Guinea-Bissau and Zimbabwe.

**Table 1:** Floristic diversity parameters and exploration level per country

Country	Observed species richness	Estimated species richness (Chao1)	Number of records	Record density/100 km^2^	Endemism %	Relative exploration	Degree of sampling completeness (from 0-1)
Angola	2262	4310 (4232–4388)	5439	0.44	18.8	0.51	0.76
Somalia	1267	2329 (2277–2381)	2892	0.45	32	0.54	0.75
Botswana	920	1378 (1342–1413)	2368	0.41	4.7	0.56	0.79
Republic of Congo	2403	3795 (3741–3848)	6439	1.88	1.5	0.63	0.82
Guinea-Bissau	841	1329 (1298–1360)	1910	5.29	0.8	0.64	0.78
Zimbabwe	2807	4165 (4113–4217)	8104	2.07	7.6	0.65	0.84
Burkina Faso	879	1309 (1280–1338)	2718	1.00	0.6	0.67	0.86
Zambia	3309	4887 (4831–4942)	11,048	1.47	7.2	0.68	0.87
Uganda	2258	3303 (3258–3348)	6818	2.83	2.8	0.68	0.86
Mali	903	1309 (1282–1336)	2550	0.21	1.4	0.69	0.84
Senegal	1342	1921 (1888–1953)	4294	2.18	4.5	0.70	0.87
Guinea	2533	3583 (3537–3630)	11,082	4.51	4.7	0.70	0.92
Rwanda	1883	2608 (2572–2644)	7740	29.39	3	0.72	0.91
Central African Republic	2560	3463 (3423–3503)	11,282	1.81	2.5	0.73	0.92
Sierra Leone	1883	2513 (2482–2545)	6470	8.95	2.3	0.75	0.89
Nigeria	3378	4487 (4443–4530)	14,907	1.61	2.2	0.75	0.92
Malawi	3340	4371 (4330–4413)	12,410	10.47	6.5	0.76	0.91
Mozambique	4095	5264 (5220–5309)	23,181	2.89	8.4	0.77	0.94
Burundi	2788	3556 (3521–3591)	12,118	43.54	2.9	0.78	0.93
Ethiopia	4481	5627 (5581–5672)	31,795	2.88	19.9	0.79	0.96
Equatorial Guinea	3049	3821 (3785–3856)	15,341	54.69	1.8	0.80	0.94
Kenya	4759	5948 (5904–5991)	28,223	4.86	11.5	0.80	0.95
São Tomé and Príncipe	806	999 (982–1016)	3598	373.24	12	0.80	0.94
Ghana	2971	3634 (3601–3667)	14,428	6.05	1.5	0.81	0.94
Dem. Rep. Congo	8860	10,872 (10,814–10,931)	111,179	4.74	18.3	0.81	0.98
Tanzania	8727	10,550 (10,496–10,605)	82,850	8.75	19.4	0.82	0.98
Ivory Coast	3689	4344 (4311–4377)	41,666	12.92	2.6	0.85	0.98
Liberia	2403	2806 (2781–2830)	18,299	16.43	3.8	0.86	0.97
Gabon	5236	6106 (6068–6144)	93,828	35.05	10.5	0.86	0.99
Benin	2460	2864 (2840–2889)	21,914	19.10	1.6	0.86	0.98
Cameroon	6883	8015 (7972–8057)	90,222	18.98	9.3	0.86	0.99

Countries are ordered from least to best botanically explored. Values calculated based on georeferenced and non-georeferenced (when the country was indicated) specimens

**Fig. 7:**
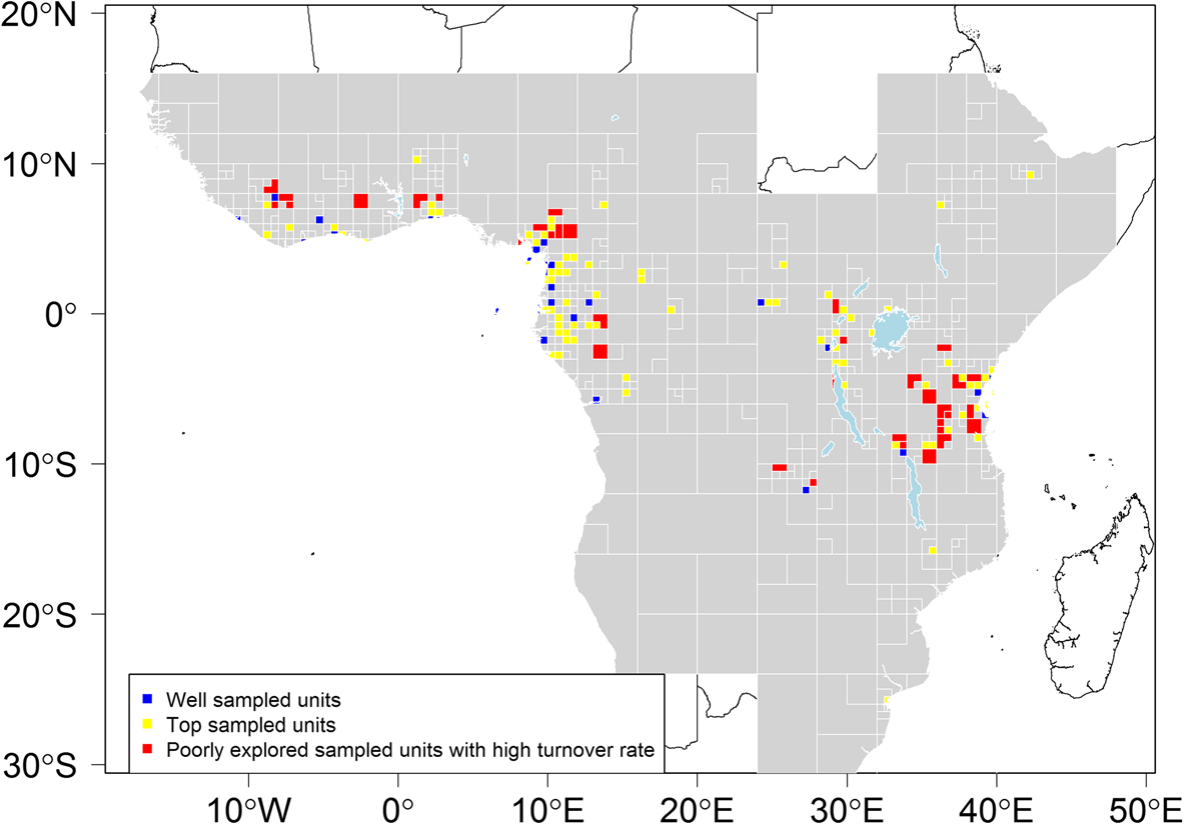
Level of botanical exploration across tropical Africa. Based on an adaptive resolution sampling units (SUs). This map shows priority SUs calculated based on a turnover rate using *μ* = 1°. Grey SUs represent SUs that did not meet any of our threshold limits (see text for explanation) and thus highlight SUs that are poorly documented. Dashed lines represent the limits for tropical Africa as defined in our study. Map based on georeferenced herbarium records, silica gel samples and plot data

Our results show that big portions of tropical Africa remain poorly explored, which is illustrated by the numerous large grey coloured SUs (areas that do not attain our threshold values for adequate botanical exploration; Fig. 7) located in, for example, Angola, DRC or Nigeria. Only 34 variable sized SUs can be qualified as being ‘well sampled’ (density above 100 records/100 km^2^ and coverage over 0.5 WSUs). These are mainly concentrated in Cameroon, Gabon and Equatorial Guinea. The TSUs are mainly located in Atlantic central Africa (Cameroon, Gabon and Equatorial Guinea), parts of Tanzania, parts of the Kivu region, and south Benin. Finally, we highlight several regions as PSUs, mainly occurring in regions in West Africa, along the Cameroon Volcanic Line, eastern Gabon, and the Eastern Arcs and coastal forests of Tanzania. It is important to underline that this does not mean that other areas are not a priority, but simply indicates that, based on our data, the exploration of these SUs is more likely to improve our knowledge about tropical African plant biodiversity.

### Growth form information

**Fig. 8:**
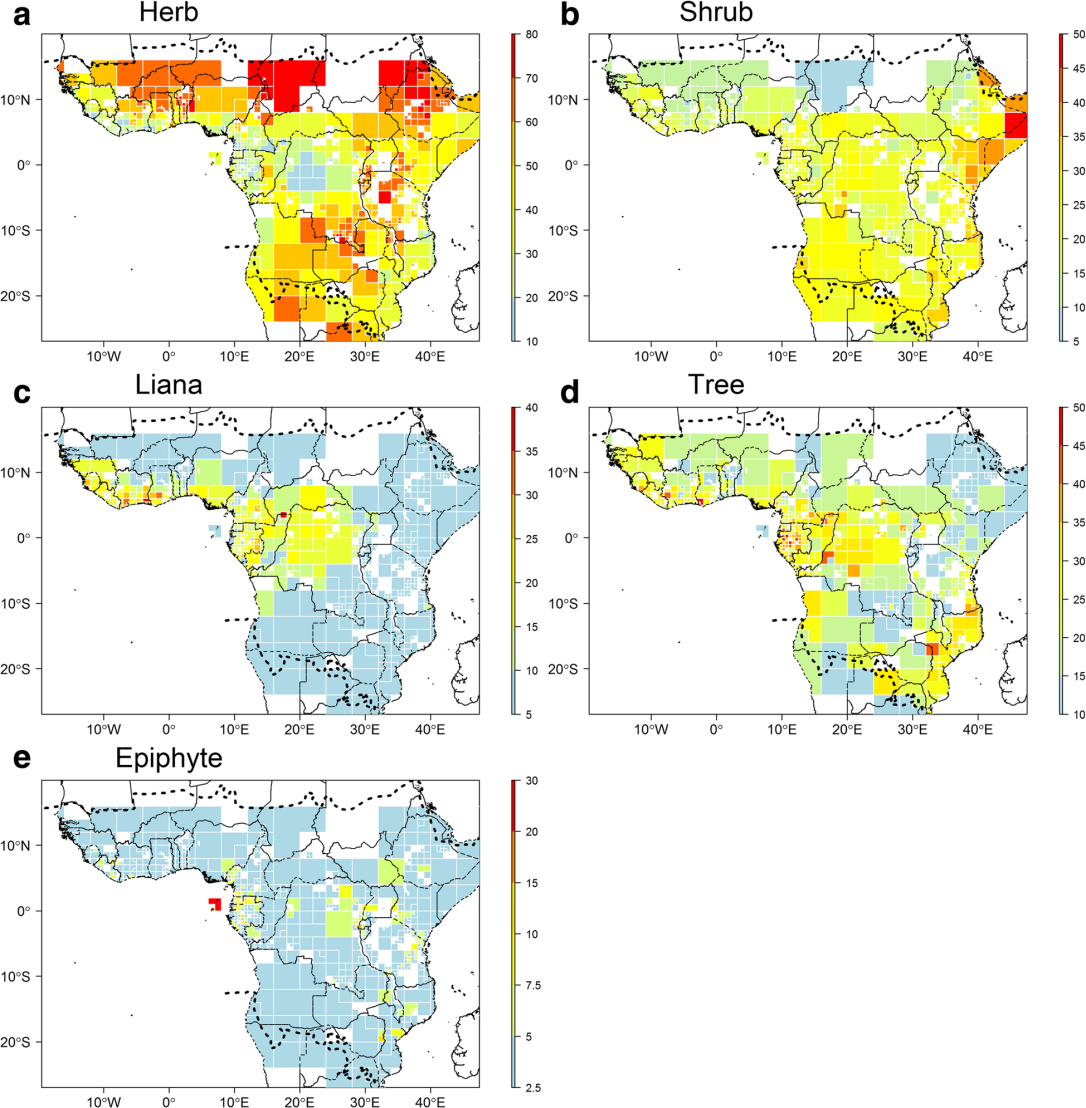
Distribution of growth form diversity across tropical Africa. Based on an adaptive resolution sampling units (SUs; for explanation see text). Proportion of species of a given growth form type occurring in each SU. **a** Proportion of herbs; **b** Proportion of shrubs; **c** Proportion of lianas; **d** Proportion of trees; **e** Proportion of epiphytes. Dashed lines represent the limits for tropical Africa as defined in our study. Map based on georeferenced herbarium records, silica gel samples and plot data

Growth form data were recorded for 21,901 species or 97% of the filtered species list of RAINBIO. Our results suggest that the number of herbaceous species is around the same as the number of woody species (Table 2). The geographic distribution of plant growth form types across tropical Africa in terms of the proportion of species of a certain type shows contrasting patterns (Fig. 8). The herbaceous growth form has a high proportion in drier regions (Sahel, East Africa) where savannah prevails (Fig. 8a). In contrast, the tree and liana growth forms have high ratios within the rain forest regions where climate seasonality is lowest (Fig. 8c, d). Shrubs recorded average proportions in the Sahel region and high proportions in the horn of Africa region, for example, Somalia and northern Kenya (Fig. 8b). Finally, epiphytes recorded high proportions in São Tomé and Príncipe, and montane areas such as the Cameroon Volcanic Line, Crystal Mountains area in northwest Gabon or Nimba Mountains in Liberia (Fig. 8e).

**Table 2:** Distribution of plant species in the RAINBIO database across growth form types

	Growth form	# species	% of total
Herbaceous	Herb	9818	46.3
	Vine	493	
	Aquatic	111	
	Mycoheterotroph	27	
Woody	Tree	3601	45.7
	Shrub	4956	
	Liana	1756	
Herbaceous/woody	Epiphyte	878	5.0
	Parasitic	261	
	Unknown	676	3.0
	Total	25,356	100%

### African tropical forest diversity

When limited to tropical African forests (Additional file 3), RAINBIO records relate to a total of 15,387 vascular plant species, of which 3013 are scored as trees, 5755 as herbs, 1637 as lianas and 3158 as shrubs (Table 3). As expected, the central African forests represent the most species rich block with 10,306 species, followed by the east African forests with 6789 species and West Africa with 4396 species. The endemism rate for Central African forests is 29.1% (2997 out of 10,306 species endemic), 7.4% for east African forests (504 out of 6789 species) and 11.4% West African forests (503 out of 4396 species). The top 20 most species-rich families found in tropical African forests are provided in Table 4.

**Table 3:** Growth form diversity across tropical African forests

		Tropical African forests	West African forests	Central African forests	East African forests
All growth forms	Total # records	383,414	72,753	260,695	46,711
	Observed # of sp.	15,387	4396	10,306	6789
	# endemic sp.	4544	503	2997	504
Tree	Total # records	122,555	19,515	92,177	10,283
	Observed # of sp.	3013	892	2264	1055
	# endemic sp.	1108	119	803	61
Herb	Total # records	97,835	19,604	60,251	16,621
	Observed # of sp.	5755	1773	3510	3277
	# endemic sp.	1078	137	560	247
Liana	Total # records	52,546	14,630	35,039	2458
	Observed # of sp.	1637	624	1384	354
	# endemic sp.	610	77	422	13
Shrub	Total # records	84,399	14,904	55,697	13,110
	Observed # of sp.	3158	664	1966	1390
	# endemic sp.	999	106	718	93

The table indicates the total number of records, total number of species and endemics observed for tropical African forests, and West, Central and East African forests separately, for all species and per major growth form type

**Table 4:** List of top 20 most species-rich plant families and genera recorded in tropical African forests

Family	Total # species	Genus	Total # species
Rubiaceae	1698	*Psychotria*	251
Fabaceae	1590	*Cyperus*	179
Orchidaceae	928	*Polystachya*	156
Asteraceae	753	*Crotalaria*	154
Poaceae	722	*Pavetta*	145
Acanthaceae	526	*Vernonia*	115
Cyperaceae	443	*Combretum*	108
Apocynaceae	430	*Begonia*	107
Malvaceae	413	*Impatiens*	102
Euphorbiaceae	402	*Indigofera*	102
Lamiaceae	367	*Euphorbia*	101
Annonaceae	306	*Rinorea*	99
Melastomataceae	231	*Habenaria*	92
Phyllanthaceae	190	*Cola*	91
Sapindaceae	176	*Dichapetalum*	89
Celastraceae	167	*Justicia*	88
Sapotaceae	164	*Asplenium*	82
Asparagaceae	159	*Millettia*	82
Convolvulaceae	148	*Bulbophyllum*	80
Polypodiaceae	141	*Phyllanthus*	79

The families and genera are ordered from largest to smallest

## Discussion

### The RAINBIO mega-database

The basis of the current analyses relies on data available through the RAINBIO mega-database [33]. As indicated in the methods section, we suggest that RAINBIO is the largest and most accurate database for tropical African plant distributions, as we undertook numerous data validation routines, automatic as well as manual via numerous African flora taxonomic experts. Recently, another dataset has been published [51], comprising 3.1 million records for 40,401 vascular plant species across the whole of Africa. However, this dataset was assembled to test a new method of identifying areas of important biodiversity conservation (Star ratings) and has not been specifically used to explore tropical African plant biodiversity.

We stress that, like all existing biodiversity databases, RAINBIO is not perfect. First, specific data associated with specimens can be erroneous, for example, misidentifications or errors in geo-referencing. Nevertheless, by having expert taxonomists validate large parts of the identifications and by treating information from duplicated herbarium specimens in a systematic manner (see recommendations section), we significantly limited errors and improved the overall quality of RAINBIO. Second, several important herbaria were not directly included such as the Muséum National d’Histoire Naturelle (P), the Conservatoire et Jardin botaniques, Geneva (G), the East African herbarium (EA) and partly the Royal Botanical Gardens, Kew (K) (acronyms follow, [11]). In general, specimens within those herbaria have not yet been databased or were not available at the time of this project. The impact of missing data has yet to be explored and could affect the results in various regions such as East Africa (EA, K) or West/Central Africa (P). However, it is important to underline that (1) a significant part of the data within these herbaria are indirectly available in RAINBIO (via duplicates distributed to other herbaria or via specimen databased with the framework of regional/country floras (e.g. [52, 53]) or monographic revisions, information of which is included in RAINBIO), and that (2) our paper explores general tropical African plant biodiversity patterns for which RAINBIO provides a sound representation of all data.

### The floristic diversity of tropical Africa

Botanical records across tropical Africa are well distributed but highly heterogeneous (Fig. 1a). Continental or regional scale biodiversity data are typically distributed in an uneven and patchy way [20, 24, 40], related to differences in accessibility (presence of roads or rivers), focus of researchers, projects [54] or to colonial history. In tropical Africa, areas of higher record density are partly explained by focused collecting and databasing efforts of the main contributing herbarium institutions (acronyms follow [11]): BRLU for Equatorial Guinea, São Tomé and Príncipe and Gabon; MO for the Eastern Arc Mountains in Tanzania and Gabon; WAG for West Africa, Cameroon, Gabon and Ethiopia; BR for western DRC and the Kivu-Rwanda-Burundi region; and K for western Cameroon. In contrast, low record density is not only due to low collecting efforts (Central African Republic, Republic of Congo or Angola) but also to incomplete digitization and georeferencing of existing records, for example, in Angola [55] and DRC [56].

For tropical Africa, we record occurrences for 22,577 species, 2810 genera and 258 families of vascular plants. These values are lower than previous estimates for the region. Indeed, Klopper et al. [2] recorded a total of 32,424 angiosperm taxa, while Govaerts [57] estimated around 29,887 species for the same region. However, these values are not entirely comparable. First, they are based on databases (checklists) of published names (e.g. African Plant Checklist and Database Project; Kew World Checklist) rather than from herbarium specimens. Due to new insights in taxonomy, a significant number of those names have been marked as synonyms in our list, although we have also added many newly published species names. Second, Klopper et al.’s [2] values are for taxa (thus including infraspecific names) and for angiosperms only, while Govaerts’ [57] are restricted to species and seed plants (gymnosperms and angiosperms). Our values are for species and all vascular plants (pteridophytes, gymnosperms and angiosperms), and based on herbarium specimens complemented with silica gel data and plot inventories. Most dubious names or names of doubtful taxonomic status still present in checklists do not have any specimens linked to them in herbarium databases.

Several species-rich areas across tropical Africa (Fig. 2a) are highlighted based on the Chao1 estimator (but see below), namely the Cameroon/Gabon forests, including Mont Cameroon, the east Kivu region and the Eastern Arc Mountains in Tanzania, with a less pronounced diversity centred in Katanga (south-eastern DRC). These results are consistent with those of several previous studies [30–32], but our results are derived from a significantly larger dataset. Larger areas with high species and specimen records can be linked to specific plant inventory efforts, for example, in most of Gabon [52], the Cameroon Volcanic Line in western Cameroon [58–60], Benin [53] and the Eastern Arc mountains [61]. Some small-scale instances of high species richness (isolated red SUs in Fig. 2a) can be either attributed to single-person sampling campaigns (e.g. in south-western Tanzania, where A.F. Stolz (1871–1917) made ca. 2500 plant collections; in south-western DRC, where Masens Da Musa generally collected each species only once [62]; or in western Ghana, with specimens mainly collected by C. Jongkind) or by focused sampling and digitization efforts for the area, namely Yangambi (North-central DRC), where intense field work was undertaken by Belgian collectors combined with the digitization of the BR type specimens and large parts of the Yangambi general collections [63]; or the Nimba Mountains area (border between Liberia, Ivory Coast and Guinea), well documented by Adam [64], with more recent records from MO and WAG contained in RAINBIO.

The estimated species richness per SU based on Chao1 (Fig. 2a) is highly correlated to both the number of records (Fig. 1a) and the observed species richness (Fig. 1b) per SU. This is a well-known artefact of biodiversity analyses [39, 40] and is likely biased by the heterogeneous sampling effort. Part of the correlations can also be explained by collector’s behaviour, tending to collect in areas of known high species richness [24, 54]. The Nielsen diversity estimator was proposed to correct for such heterogeneous sampling effort [39]. When computed for each 0.5° and 1° SUs (Fig. 2b), this effective number of species is much less correlated with specimen density (Pearson correlation *R* = 0.28). Despite that high species diversity is confirmed in Lower Guinea and the Eastern Arc Mountains, there are some discrepancies between richness patterns shown by the Chao1 and Nielsen estimators. For example, estimated species richness based on Chao1 in the Kivu and Katanga regions of DRC and several areas in Lower Guinea are similar, while this pattern is not observed when using the Nielsen estimator. This suggests the Chao1 does indeed more strongly reflect the heterogeneous sample size rather than the true species diversity. Other differences are the high effective number of species observed in several areas in Ethiopia and West Africa, localities that were not emphasised by the Chao1; it is likely that these are artefacts in the Nielsen estimator. For example, the very high effective number of species detected in western Ghana (red SU, Fig. 2b) is probably due to the intense collections of a single collector (C. Jongkind) in that area. Large parts of Gabon yield a very low Nielsen score, which must be a flaw in the Nielsen estimator, since several SUs were identified as well as top sampled units (WSU or TSU; Fig. 7), and our data (Fig. 1b) confirm they are very species rich. It is nevertheless important to highlight that the Nielsen and Chao1 estimators are not fully adequate for herbarium specimens because they rely on the relative abundance of species within each SU (even if only singletons and doubletons are taken into account for Chao1 estimator), which is estimated by the number of specimens per species. However, the number of specimens per species is not a good proxy of the species’ population size [54]. Indeed, a collector generally does not collect plants at random, but usually tends to collect as many different species as possible. This would explain the unexpected high values of Nielsen estimator and Chao1 observed in SUs where many specimens were collected by a single or very few collectors. The SU in western Ghana with mainly collections by C. Jongkind does give an artefact in Nielsen, but not in Chao1, indicating that such collector-biases are not dealt with in the same way.

### The diversity of tropical African forests

Our study documents a total of 15,387 vascular plant species occurring in tropical African forests (excluding Madagascar) of which ca. 30% are strict endemics (Table 3). To date, only rough estimates have been published, and these have generally not been restricted to forested regions but rather to certain major bioregions such as phytochoria [3, 31, 65]. White [26] estimated a total of 8000 plant species occurring in the Guineo-Congolian regional centre of endemism, 80% of which were endemics. This centre of endemism concerns West and Central Africa and encompasses species occurring in other ecosystems than forests such as savannahs and mountains. The values advanced by White [65] are based mainly on his expert opinion with little underlying quantitative information.

Adding to previous estimates of the tropical tree flora [20, 27], our results suggest a total of 3013 tree species recorded for African forests (36% strict endemics). This estimate is below the recently estimated minimum number of 4626–5984 trees for Africa [27], but it provides, for the first time, a number based on solid underlying quantitative biodiversity data. However, the values of Slik et al. [27] include Madagascar and are based on forest plots where a tree is identified as having a diameter above breast height (DBH) of more than 10 cm. Recent studies have underlined that a significant portion of tree species diversity in African forests have a DBH smaller than 10 cm [66, 67]. In our analyses, a species was considered a tree if explicitly mentioned on the specimen labels or coded as such [33], thus also includes species with DBHs smaller than 10 cm.

Our results confirm the observed ‘odd man out’ pattern [29] of relatively lower diversity of African forest tree species when compared to the Neotropics or South-East Asia [20, 27, 28, 68]. For example, there are 11,676 tree species (DBH > 10 cm) recorded for the Amazon basin, more than three and a half times our figure for tropical Africa. Overall, the tropical African forest tree flora represents between 5% and 7.5% of the estimated total number of tree species for the whole of the tropics (between 40,000 and 53,000 tropical tree species, [26]). The numbers documented here, though likely underestimates, provide for the first time values for plant diversity in tropical African forests. As indicated above, significant discrepancies between ‘true’ floral diversity and estimated/counted number of species in a given region is a common problem in the tropics, as rare species will be hard to find or document [20].

### Country-level diversity patterns

We provide, for the first time, basic plant biodiversity values for several countries across tropical Africa (Table 1). These values are based on the available data within RAINBIO, and thus should not be taken as definite. Nevertheless, they provide important insights into the plant diversity of these countries as well as levels of exploration. For some countries, these values are quite close to previous estimates. For example, Onana [69] recorded a total of 7850 vascular plant species for Cameroon, whereas we report 6883 species with an estimated total species richness of 8015. The higher recorded total species number of Onana [69] could be related to synonymy not taken into account during that study (Onana personal communication). Another example is for São Tomé and Príncipe [70], where a total of 803 native flowering plant species (excluding 301 introduced species) plus a single endemic gymnosperm species have been recorded. This value closely matches our estimates (Table 1), which could reflect the inclusion of the vast majority of São Tomé and Príncipe records from herbaria such as BRLU and LISC in our study [33].

Our study identifies several botanically ‘best-explored’ countries with Cameroon, Benin and Gabon in the top three (Table 1). For some of those countries (i.e. Benin, Gabon and Liberia) a large percentage of existing specimen data (over 90%) is available in RAINBIO and thus could reflect values close to reality. Even for these countries, the average specimen density is well below the threshold of 100 records/100 km^2^ (19 for Benin, 35 for Gabon and 16 for Liberia; Table 1). In addition, the estimated discover rate (percentage of species yet to be documented based on our estimate of total species richness) varies between 14% and 19% for the top seven countries (Table 1). Thus, even for the top best-explored countries new species are expected to be described or have already been collected but not yet identified. Data from the International Plant Name Index (IPNI, www.ipni.org) for the period 2000 to 2015 reveals that 8162 and 38 new species were described from Benin, Gabon and Liberia, respectively (0.3%, 2.7% and 1.5% of their estimated species richness, respectively). For Gabon, 4710 species were recorded in 2005 [52], meaning that no less than 526 (10%) new species or new species records have been added these past 11 years (5236 species recorded in RAINBIO). New species and even new genera are still regularly described for Gabon, even in well sampled areas [71] such as the Crystal Mountains National Park [67, 72–77]. Interestingly, São Tomé and Príncipe show the highest concentration of records (375/100 km^2^, Table 1) yet is not amongst the best-explored countries in our study. This could be linked to the relatively high level of singletons when compared to the other most “well-explored” countries, with 233 species known from a single record out of 3598 total records (6.5%, Table 1). The Chao and Jost [45] estimator for the degree of sampling completeness adds DRC and Tanzania to the list of best-explored countries (Table 1). However, this seems counterintuitive when looking at the high number of singletons for those countries and the difference between the observed and estimated number of species (Table 1). This can be explained by the high number of specimens available for these two countries, which reduces the effect of the high number of singletons. Importantly, our results underline that even though these countries might appear well explored, there are still lots of efforts to provide in terms of botanical exploration and databasing. This is a common situation across the tropics, for example, in South America [20], where for even some of the best collected countries such as Ecuador, significant collecting efforts remain to be done in order to fully record its floral diversity [40]. To date, as far as we know, no tropical country (except Singapore) has yet claimed it should be considered as being botanically well-known.

In contrast, for some countries our data is far from complete as underlined by the low values of degree of completeness such as Angola, Somalia, Botswana, Republic of Congo, Guinea-Bissau and Zimbabwe. For some countries, the low value is explained by a lack of available data in digital format. For example, Figueiredo et al. [55] mentioned the occurrence of 6735 species of vascular plants in Angola, whereas RAINBIO records only 2262 species (Table 1). In the same way, the Republic of Congo is suggested to contain 4538 vascular plant species [78, 79], whereas our dataset records just 2403 (Table 1). Despite this basic check-list, the Republic of Congo remains botanically one of the least known countries in tropical Africa [79]; a result that is also confirmed here (Table 1).

### Botanical exploration of tropical Africa

A vital question in biodiversity inventories has been to evaluate the level of botanical exploration across the tropics. Campbell and Hammond [46] suggested that a minimal level of botanical exploration of a tropical region should be at least 100 specimens per 100 km^2^. Our data indicate that just 21 of the 0.5° SUs reach this threshold (Additional file 4, 0.5° represents a surface of about 3000 km^2^ around the equator, thus 3000 specimens per SU). In addition, we find that just 34 variable sized SUs (Fig. 7) are well sampled (defined as SUs with record density higher than 100 records/100 km^2^ and *C*_*N*_ > 0.5). This is much less than the number identified by Stropp et al. [25] based on the analysis of GBIF data (1002 SUs of 0.25° with more than 200 records). However, most of these SUs were located in South Africa, a region not included in our study. Moreover, this difference could be linked to the data (RAINBIO versus GBIF, although RAINBIO contains more data for tropical Africa) or the difference in our definitions of well-sampled SUs (larger SUs (0.5° versus 0.25°), different density thresholds and a different method to calculate the sampling coverage, *C*_*N*_). In addition, we defined TSUs, which highlight the 25% SUs with the best data available when compared to the others (Fig. 7). TSUs are different from WSUσ in that they show SUs where most botanical exploration has occurred relative to others. Finally, the need for additional botanical exploration is also reflected by the very high number of species known from less than five records (Additional file 5a) or occurring in a single SU (Additional file 5b); this is a characteristic outcome of tropical biodiversity inventories [52, 80, 81]. For our dataset, this can partly be explained by the targeted scanning of type specimens within the Global Plant Initiative ([63], https://plants.jstor.org), artificially increasing the number of species known from a single collection. Still, overall, our results confirm that tropical Africa remains severely under sampled [24, 25], even when using the largest homogenised dataset ever complied for the region to date [33]. Similar results are reported for other tropical regions such as the Amazon basin [20] or countries such as Ecuador [40].

In a world of limited resources, an important question is how to identify priority sampling units [24], e.g. SUs where we could expect to significantly add to our knowledge of the African flora. This is not a trivial question, as potentially any SU across Africa deserves more in depth exploration. Several past studies have identified data-deficient areas using different methods. Küper et al. [24] qualified data-deficient areas as the difference or the ratio between predicted species richness (based on species distribution modelling of ca. 5000 species) and the documented species richness. Stropp et al. [25] identified areas of ‘acute data deficiency’ as areas that maximise the distance between well-sampled SUs based on the assumption that floristic similarity decreases with distance. Both these approaches are valid in their own right, underlining regions either containing potentially many uncollected species or regions that maximise the collection of new records for a whole region, respectively. Here, we used a concept similar to Stropp et al. [25] to identify a set of PSUs. Indeed, floristic similarity does decrease with distance; however, this relationship is not linear, as certain regions will experience higher floristic turnover rates than others (Fig. 6) [32]. We suggest that regions of higher floristic turnover should be more thoroughly explored as they will potentially uncover higher levels of botanical novelties. We defined a PSU as being an SU with an estimated high turnover rate relative to other neighbouring SUs associated with a low sampling coverage. High floristic turnover rates were identified (Fig. 6) mainly in montane areas (Cameroon Volcanic Line, Nimba Mountains, Eastern Arcs) and areas of vegetation transitions (e.g. coastal regions in West, Central and East Africa, Katanga in south-eastern DRC, Haut-Ogooué in Gabon). Not all coastal regions are identified as having a high turnover (e.g. Liberia), but the high turnover observed along the southern coast of Gabon is corroborated by Harris et al. [82]. Our suggested PSUs (Fig. 7) highlight very different areas than those suggested by Stropp et al. [25] and agree more with the priority areas suggested by Küper et al. [24]. Indeed, we identify PSUs, for example, in Guinea (Nimba Mountains), Cameroon (the Volcanic Line area above Mount Cameroon and the northern part of Korup National Park), Gabon (Haut-Ogooué), and the Eastern Arc Mountains and coastal forests in Tanzania. Areas of low estimated floristic turnover (Fig. 6) [32], such as the Congo Basin, do not contain any PSUs, in contrast to Stropp et al. [25]. Even though one could criticise our approach as it relies on the estimation of turnover rates, we applied a variable SU size approach which allows comparison of SUs based on similar amount of data [35]. All approaches to identify PSUs can be viewed as complementary but focusing on different aspects/priorities of biodiversity exploration.

### Plant growth form dominance across tropical Africa

Data on growth form in the tropics is scarce, but some studies show that the contribution of herbaceous species to tropical forest diversity is between 18% and 44% [83–85]. Our data suggests that 43.8% of species are herbaceous across Tropical Africa. This also underlines that savanna and montane vegetation types are well represented in RAINBIO, despite the initial emphasis of our data towards forested regions. It has been shown that on global or continental scales plant growth form is linked with climatic variables [19, 86, 87]. Although no formal analyses are undertaken here, our data underline general patterns possibly linked to climate (Fig. 8). For example, herb species are dominant in the drier parts of Africa (Senegal, Burkina Faso, Malawi, Tanzania, Zimbabwe) whereas trees and lianas are dominant in countries with significant areas of tropical forests (e.g. Republic of Congo, Gabon, south of Cameroon). In addition, with most of their surface being at higher elevations, Ethiopia, Rwanda and Burundi harbour vegetation with much higher proportions of herbaceous species.

As indicated above, the herbaceous component (Fig. 8a) gives a clear pattern related to the drier regions (Sahel, East Africa) where savanna prevails. Rwanda, Burundi, south-eastern DRC (Katanga), Zambia and Ethiopia, which are dominated by savanna and/or highland vegetation, also stand out as containing a high proportion of herbaceous species. Interestingly, a large New World dataset for geographical trait patterns did not reveal montane areas (such as the Andes or the Rocky Mountains) as exceptionally dominated by herb species [19]. The moderately high ratio of herb species locally observed on the coast of Ghana and the western tip of Gabon (Port-Gentil) (Fig. 8a) relate to land surface occupied by coastal savanna where the rest of those grid cells are sea, showing that marginal SUs with only little land surface can easily result in seemingly odd results.

Shrub dominance (Fig. 8b) is in general more equally distributed than herb, liana and tree dominance; shrubs can occur in nearly all vegetation types. The Eastern Africa Coastal Forest ecoregion is strikingly dominated by shrubs, known for its mosaic of forest, savannas and wetlands [88–90]. High shrub dominance is also observed in the Horn of Africa. The low proportion of shrub species observed in montane areas is not corroborated by a study on New World plants [19].

Liana dominance (Fig. 8c) shows a very clear pattern, being high within the major rain forest block (the Guineo-Congolian region). In addition, lianas form a high proportion of growth forms in the semi-deciduous forests of south-eastern Cameroun and south-eastern Gabon, as well as in the highly fragmented forests of Upper Guinea [91], while the wettest forests in coastal Gabon, Cameroon and Liberia contain proportionally fewer liana species. This might be explained by the wettest forests having a closed canopy all year round, rendering the understory very dark, while in the drier forests, with some of the trees being semi-deciduous, the forest floor will have some periods with more light, enabling liana seedlings to gain the energy to climb to the canopy. In contrast to west and central Africa, the drier semi-deciduous East African coastal and Eastern Arc forests contain proportionally fewer liana species, which remains unexplained.

The tree component (Fig. 8d) is highest within the rain forest regions where climate seasonality is lowest, which thus is in line with the findings of Engemann et al. [19] for the Neotropics. Gabon, having over 80% of its surface covered by possibly the most species-rich lowland rain forest of Africa [92], shows the highest proportion of tree species and the lowest proportion of herb species. Interestingly, similar high dominance in tree species is observed in northern Mozambique and south-eastern Tanzania. Recent collections from that area were made in the framework of the tree flora of Mozambique [93], creating a potential bias towards tree records. The Katanga region (south-eastern DRC) comes out as an area with an extremely poor tree species component.

Overall, the proportion of epiphytes (Fig. 8e) is highest in montane areas such as Nimba Mountains (northern Liberia), Mount Cameroon and the Bamenda Highlands (Western Cameroon), Crystal Mountains (north-west Gabon), Monte Alen (Rio Muni), the Albertine Rift region and the Eastern Arc Mountains. Orchids, which constitute 73% of all epiphytes species recorded in RAINBIO, having better dispersal abilities than most other groups, would have been favoured by elevation and humidity gradients in mountainous areas [94, 95]. The exceptionally high proportion of epiphytes observed on the island of Príncipe (up to 30% of species) is probably biased by the activities of several collectors with a strong interest in orchids [96]. Certainly, many more areas are actually under-collected for epiphytes, which are generally difficult to reach in the canopy.

### Collecting history

Collecting intensity steadily increased through time in tropical Africa at least up to the end of the 20th century (Fig. 4a). This trend, also underlined by other studies [25], could be linked to the growing notion of the importance of our environment and its diversity for economic reasons as well as the well-being of mankind and our planet [97]. We can clearly see the effect of both world wars (1914–1918, 1939–1945; Fig. 4a), where collecting intensity dropped, but then quickly increased afterwards. The lower number of specimens recorded for the 21st century is worrying but could be partly explained by a lag in specimen digitization [25]. However, we do believe this observation to reflect a true trend. Thus, even though our data shows that our knowledge of tropical botanical diversity is far from sufficient (Fig. 7), we observe an alarming trend of diminishing exploration efforts across tropical Africa (Fig. 4a). We also identified two decades (1941–1950 and 1961–1970) where significant botanical collecting took place in SUs never visited or very poorly collected before (reflected by the negative slope in Fig. 4b). For 1961–1970 this is indeed explained by a record newly inventoried SUs (Additional file 7). Whereas for the 1941–1950 decade, this trend remains unexplained as comparatively fewer new SUs were inventoried.

When we study the geographical distribution of collecting efforts through time (time lapse) in tropical Africa (Fig. 5), we note that, until 1900, important collecting activity took place notably in Sierra Leone, the coasts of Cameroon and Gabon, in DRC along the Congo River and its main tributaries, and in East Africa in the Mombassa to Dar-es-Salaam coastal region. These corresponded to relatively easy accessible areas along the coast lines or along major river systems. Then, from 1900 to after the Second World War, the most significant botanical explorations took place in Liberia, Ghana, DRC and Mozambique. This was followed, between 1945 and 1975, by intensified collecting in virtually all regions, but notably in Ivory Coast, Cameroon, Gabon, the Albertine Rift region and Ethiopia. Finally, in the period after 1975, several regions were explored for the first time, such as northern Benin, while gaps were filled notably in Cameroon, Gabon, Kenya and Tanzania. It also is apparent that, in certain areas, there was no collecting at all, often due to periods of war (e.g. Liberia 1980–2000 and Angola 1975–2002), or the clear drop in collecting in DRC after independence (1960) when various Belgian institutions stopped their explorative work.

In general, we observe that collecting efforts per country are linked with both the political situation in a region as well as specific Flora programs. Each country has its own particular collecting history (Additional file 6). For Benin (Additional file 6a), collecting activities greatly increased in the 1990s and 2000s related to a program to support their National Herbarium and publish a diagnostic Flora for the country [53]. In Cameroon (Additional file 6b), collecting has been intense and fairly stable from the 1960s onward, initiated by the activities of René Letouzey (1918–1989) [98]. The graph for DRC (Additional file 6c) shows the significant efforts by Belgian botanists during the colonial period 1890–1960 (despite the fact that the data on the majority of these collections is not yet available in electronic form), during which the production of the *Flore d’Afrique Centrale* started [56, 99]. This is followed by a rapid decline in collecting activity after the country became independent in 1960. For Gabon (Additional file 6d), a steep increase in collecting efforts coincides with the start of the intensive research program by the Plant Taxonomy department of Wageningen University (The Netherlands) in the early 1970s, also attracting other research groups in the course of time [52]. This provided the baseline data for the production of the Flore du Gabon [100]. For Ivory Coast (Additional file 6e), the vast majority of collections were made after World War II, undertaken by local botanists (e.g. L. Aké Assi 1931–2014) but also British, French, Dutch and Swiss researchers [101, 102]. Collecting intensity dropped considerably after the turmoil in the 2000s. Collecting in Liberia (Additional file 6f) increased due to the efforts of Dutch collectors during the 1960s and 1970s. Then, after a period of virtual inactivity and civil war, renewed efforts took place, mainly by a single Dutch collector (C. Jongkind) within the context of various conservation projects and environmental impact studies [103]. Finally, Tanzania (Additional file 6 g) shows a steady increase in collecting effort after World War II, mainly by Tanzanian, American, British and Scandinavian botanists and also in the light of the production of the Flora of Tropical East Africa [61].

To conclude, we provide three main recommendations in order to improve our understanding of the distribution of plant diversity in tropical Africa.

1. *Improve data exchange between datasets*
  The effort to combine several big datasets as well as more non-public specific ones has been a major undertaking [33]. It has proven to be possible, not overly complicated, though fairly time consuming. For tropical Africa, downloading available data from GBIF will exclude significant records [25] mainly from non-public databases from institutions that are either not participating in GBIF or have not yet shared all their data openly. We did not undertake detailed comparisons between GBIF and RAINBIO data as this was out of the scope of this article. Even though GBIF represents the most important source of open data for species distribution information, it has been shown that data quality and reliability may be low, especially in the tropics [104, 105]. When compiling specimen data from different sources, one is faced with the ‘duplicate problem’, since plant specimens have several duplicates distributed to different herbaria some are prone to having different identifications and databasing quality, complicating the concatenation of data. Estimates suggest that just under 10% of all records in GBIF are potential duplicates [106]. Taking into account duplicates when compiling data from different institutes has yet to meet a good automatic solution and has rarely been done in large plant datasets. In the case of RAINBIO, different datasets were identified as ‘expert taxonomic benchmarks’, i.e. datasets verified by specialists for a given family, against which all duplicates were compared too and updated. We identified 11% of records with at least one duplicate. Similar approaches to deal with this problem have been undertaken, but at smaller scales (e.g. [105]). In order to maximise the usefulness of botanical biodiversity data more efforts should focus on resolving this limitation and finding appropriate automatic ways of taking duplicate information into account, especially from important online repositories such as GBIF that contain potentially high levels of duplicated information. Thus, besides past and ongoing major efforts to independently database herbarium specimens and upload them to GBIF, synthesising these datasets must remain a priority.
2. *Improve data reliability*
  Separate, but related to the above point, the reliability of the data is important as it will directly influence the outcome of analyses [12, 20, 104, 105]. It is well known and understood that natural history specimen datasets have several potential errors [20, 54, 107]. Two major sources of errors can influence the analyses of such data.
  First, wrong species identification of specimens will be an important source of error in any dataset. We estimate that the identification error rate in an average herbarium collection can be as high as 10% or even up to 58% for specific groups [108], although in the latter figure specimens with a synonymous but otherwise correct name were also counted as errors. In a dataset of 4000 individual trees of the genus *Inga*, 7% of the identifications proved to be wrong [109]. By comparing an expert selection of identified duplicates, the identification accuracy in combined botanical datasets like ours can be improved. However, misidentifications are an inherent part of any biodiversity dataset and can never really be completely eliminated at least because of changing taxonomic concepts and the presence of incomplete specimens. The event of DNA barcoding [110] could provide added value to accurately identify specimens (from sterile vouchers to species complexes) although bulk barcoding of large collections remains expensive and time-consuming. We must also consider the level of our taxonomic knowledge for tropical Africa. Every year, hundreds of new plant species are being described (globally, on average well over 2000/year according to data provided by IPNI, www.ipni.org), and new taxonomic borders for species, genera, etc. are drawn. Bebber et al. [9] showed that the majority of newly published species names were based on specimens collected long before. Thus, a fair number of new species are awaiting description within one of the world’s herbaria. In the future, our advancing knowledge will further refine and improve the quality analyses such as the ones presented above.
  In addition, reliability of the data can be improved by a structured collaboration continuing to work with taxonomic experts. In the case of RAINBIO, large numbers of records were indeed checked and validated by the project taxonomic experts from numerous families or geographic regions. This greatly improved the accuracy and quality of RAINBIO. Misidentification is sometimes considered as ‘background noise’ in ‘big data’ datasets. Depending on the objectives of the study this can be misleading, leading to erroneous results (e.g. when estimating total species number per country/region; conservation assessments). We advocate a ‘compile and check’ rather than a ‘compile only’ approach. Directly involving expert plant taxonomists in such large projects will greatly improve our databases and the subsequent biodiversity analysis. Second, specimens may be incorrectly georeferenced, which in turn affects biodiversity analyses [105]. However, recent tools or software packages are now available to automatically improve or correct wrong georeferencing in big datasets [111, 112]. Checking georeferencing precision using such methods should systematically be used in order to improve the precision of these records.
3. *Continue the botanical exploration of tropical Africa and digitization of the related specimens*
  No country in tropical Africa can be regarded as botanically well explored. Larger areas with no or limited data are still plentiful. Given the observed declining trends in collecting efforts (Fig. 4a) [25], we appeal not only for additional collecting efforts, but also for increased digitization of tropical African plant collections. This will depend on the availability of major funding, mostly at governmental level. In tropical Africa, the major gaps in availability of digital specimen data are in Nigeria, the Central African Republic, South Soudan, the Republic of the Congo and Angola. We believe these to be true gaps, and therefore regions for which comparatively low numbers of specimens have been collected to date. The gap in the availability of data from the DRC should at least partly be overcome soon due to major digitization efforts at BR [56]. Here, we highlight several regions (namely PSUs) we believe would provide a significant amount of new data to our understanding of the tropical African flora in the same spirit as other studies [24, 25].

## Conclusion

The RAINBIO database provides the underlining data to assist in advancing phytogeographical, systematic and ecological research, and enables a wiser sustainable usage and conservation of Africa’s unique tropical plant diversity. Only when provided with sound and high quality information on biodiversity distribution, can policymakers take informed decisions about how to manage this fragile resource effectively [8]. Strangely enough, its strength is also in that it shows clear weaknesses in the amount of botanical data available for tropical Africa. To most efficiently fill the gaps and speed up the work, we should investigate how we might benefit from a much larger group of data collectors. The development of citizen science [113], i.e. public involvement in science, has proven its usefulness in a range of biodiversity-related projects (for two recent examples, see [114, 115]). For specific plant groups, field observations made by citizen scientists backed-up with photographs and verified by specialists can add valuable distributional data especially in data-deficient areas (e.g. [115]). These ideas contrast strikingly with the observation of decreasing collecting efforts in the past two decades. In tropical Africa, the paucity of reliable data on a group of organisms as important as vascular plants cries out loud for a renewed and probably unprecedentedly massive botanical exploration of the region.

## Additional files

Additional file 1: Two examples of turnover rate calculation. The figure shows two examples to estimate the turnover rate used herein. For *μ* = 1° (meso-scale) and *μ* = 2° (large-scale). The focal sampling unit (SU) is highlighted in red. A circle of distance *μ* is drawn around the red SU. All SUs included in the circle and with record number above 100 are then selected (in grey). In white, non-selected SUs. The geographical distance between all selected SUs is then calculated based on the centroid of the convex hull around the records for each SU (not shown). The pairwise floristic similarity between all selected SUs is then computed as 1–β_sim_. The linear relation between the geographical distance and the floristic similarity between all comparisons is computed (line in red). The distance (in kilometres) that halves the initial floristic similarity is calculated (vertical line) and used to define the turnover rate for each SU. (PNG 917 kb)
Additional file 2: An R script used to compute Nielsen estimator from a matrix of samples-species. (R 808 bytes)
Additional file 3: The distribution of tropical African forests. Map showing the 0.1° sampling units selected as forest for our study based on the map of Mayaux et al. [116]. Light-green: west African forests; deep-green: central African forests; medium-green: east African forests. (PNG 136 kb)
Additional file 4: The number of 0.5° sampling units (y-axis) containing a specified number of observations (x-axis). (PNG 12 kb)
Additional file 5: Geographic distribution of records across tropical Africa. (a) The number of species known from a particular number of records. (b) Number of species known from a particular number of 0.5° sampling units. (c) Number of species in function of their calculated range size (convex hull). (PNG 1422 kb)
Additional file 6: Collecting history per country. Bar plots for a selection of tropical African countries showing the number of records collected in each period of time (5-year intervals). Plots based on herbarium records. (PNG 2657 kb)
Additional file 7: Temporal exploration of tropical Africa per decade. The figure shows the newly explored 0.5° sampling units per decade. (PNG 21 kb)
Additional file 8: A GIF animated figure of Fig. 5 by 10-year time slices. Also available at http://rainbio.cesab.org/. (GIF 2259 kb)
