# Supplementary figures and images for: Exploring the floristic diversity of tropical Africa
Source: BMC Biol. 2017 Mar 7;15:15. doi: 10.1186/s12915-017-0356-8 (PMC5339970; doi:10.1186/s12915-017-0356-8)

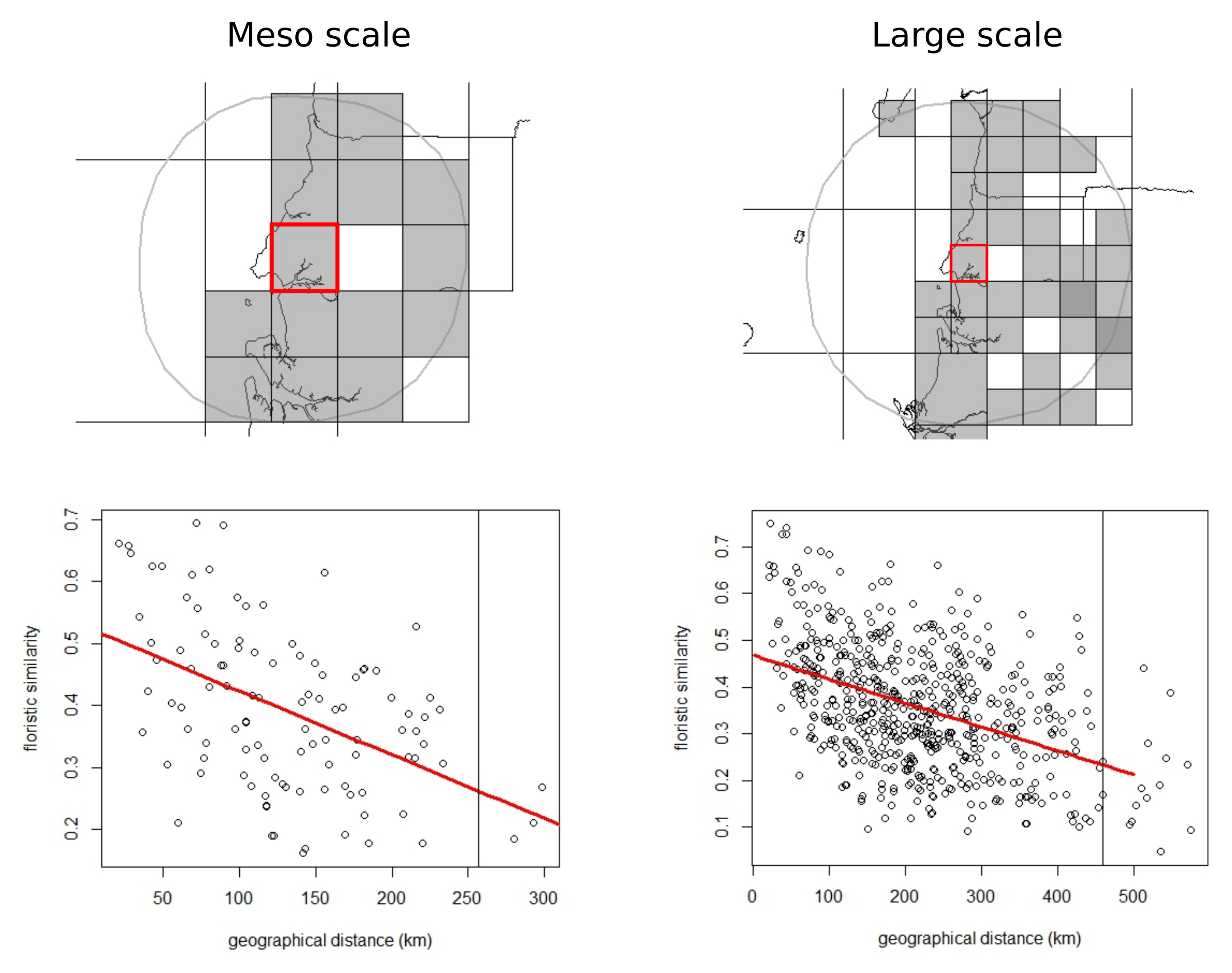

Supplement: Additional file 1: — Two examples of turnover rate calculation. The figure shows two examples to estimate the turnover rate used herein. For μ = 1° (meso-scale) and μ = 2° (large-scale). The focal sampling unit (SU) is highlighted in red. A circle of distance μ is drawn around the red SU. All SUs included in the circle and with record number above 100 are then selected (in grey). In white, non-selected SUs. The geographical distance between all selected SUs is then calculated based on the centroid of the convex hull around the records for each SU (not shown). The pairwise floristic similarity between all selected SUs is then computed as 1–βsim. The linear relation between the geographical distance and the floristic similarity between all comparisons is computed (line in red). The distance (in kilometres) that halves the initial floristic similarity is calculated (vertical line) and used to define the turnover rate for each SU. (PNG 917 kb) [file 12915_2017_356_MOESM1_ESM.png]

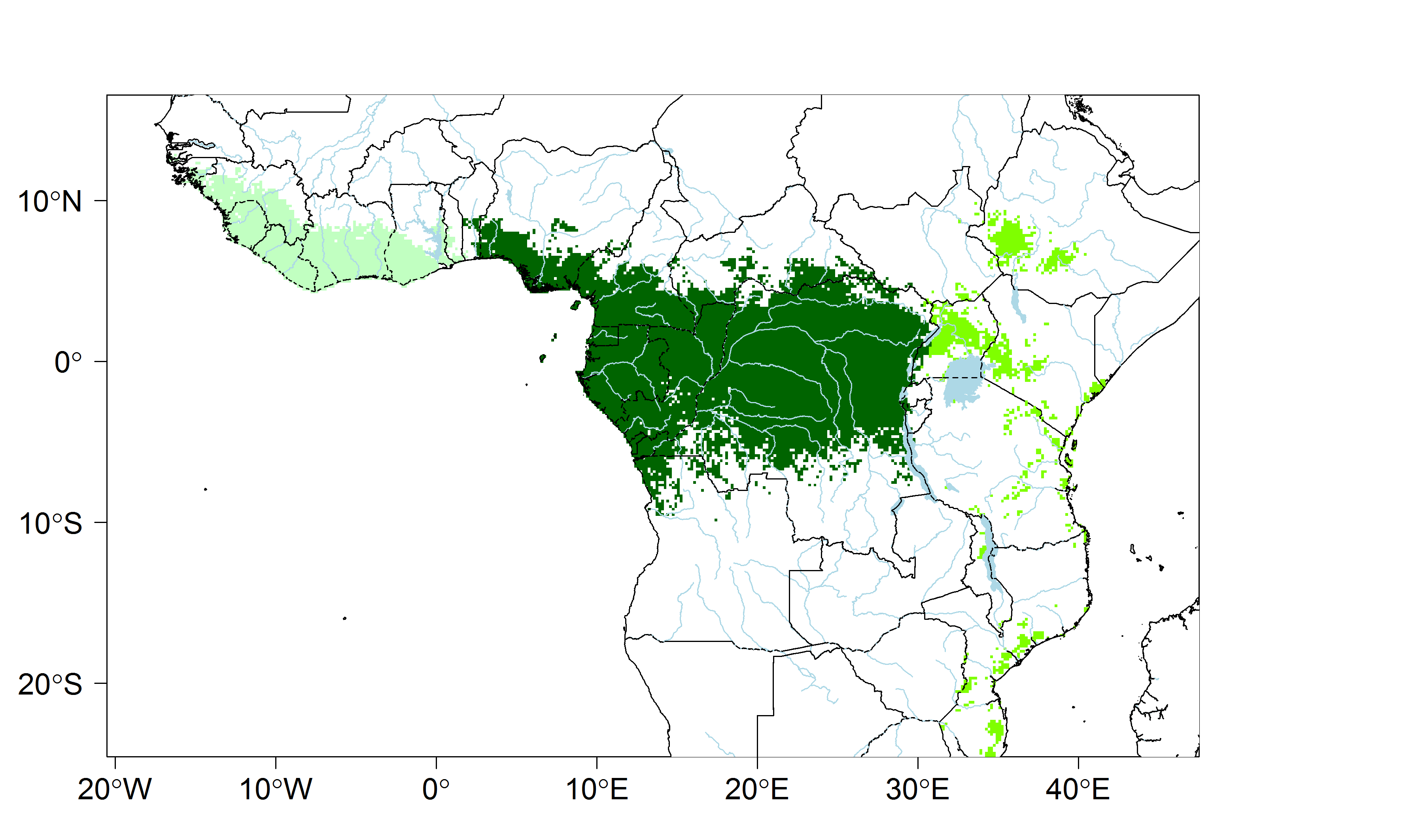

Supplement: Additional file 3: — The distribution of tropical African forests. Map showing the 0.1° sampling units selected as forest for our study based on the map of Mayaux et al. [116]. Light-green: west African forests; deep-green: central African forests; medium-green: east African forests. (PNG 136 kb) [file 12915_2017_356_MOESM3_ESM.png]

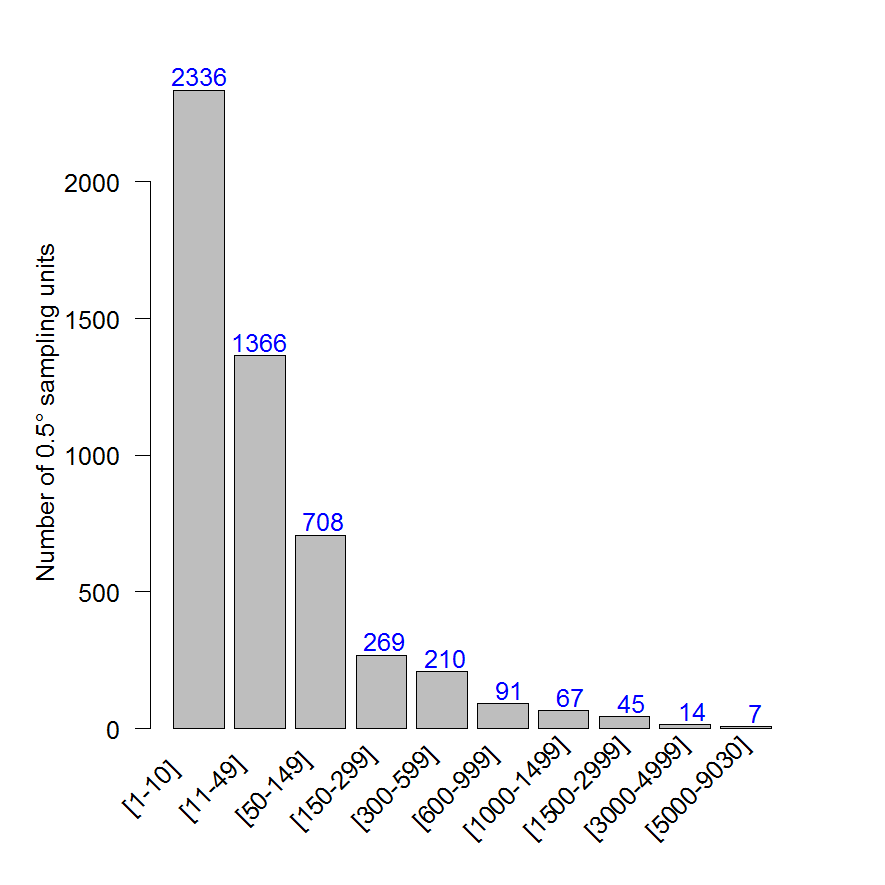

Supplement: Additional file 4: — The number of 0.5° sampling units (y-axis) containing a specified number of observations (x-axis). (PNG 12 kb) [file 12915_2017_356_MOESM4_ESM.png]

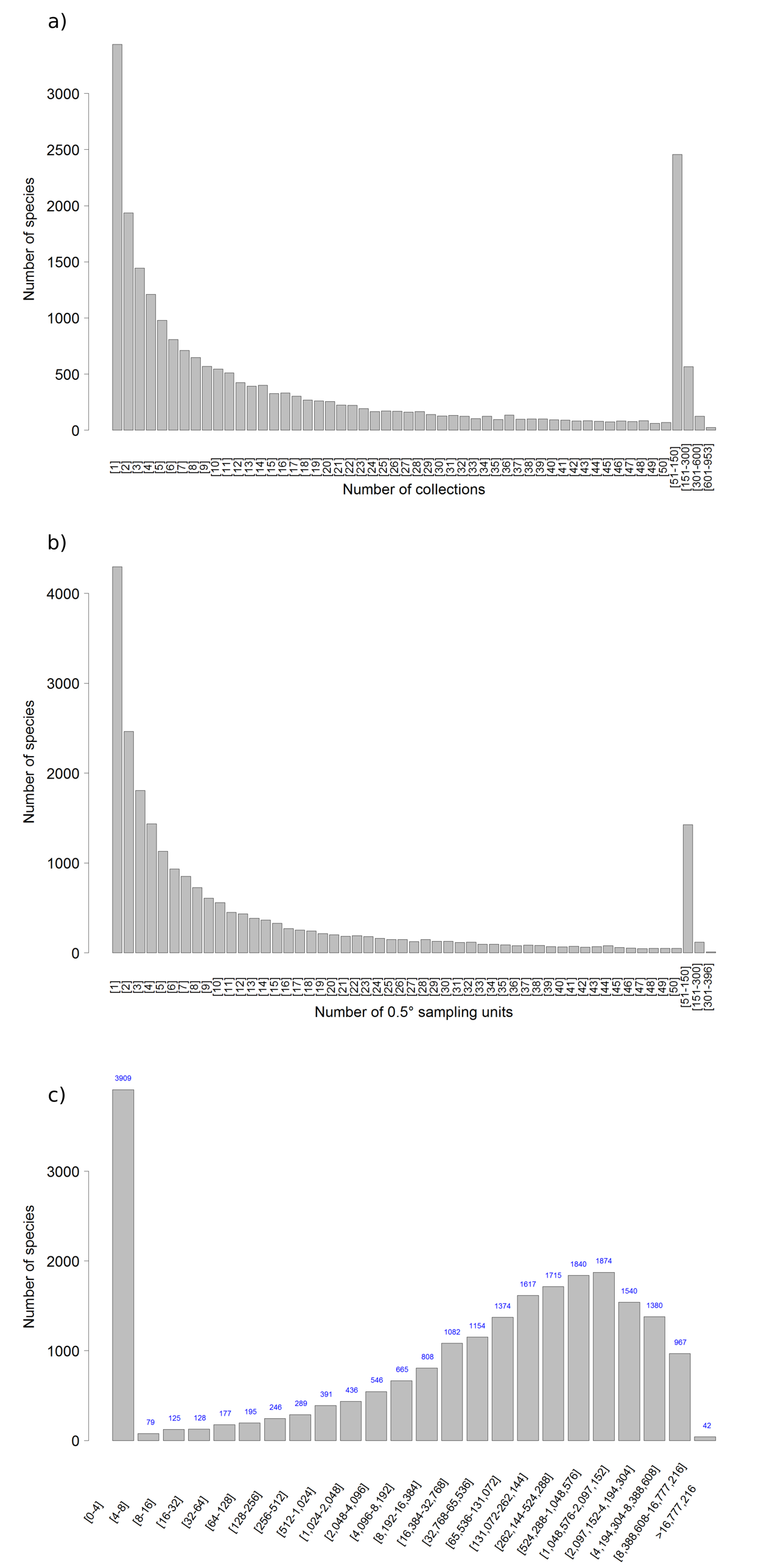

Supplement: Additional file 5: — Geographic distribution of records across tropical Africa. (a) The number of species known from a particular number of records. (b) Number of species known from a particular number of 0.5° sampling units. (c) Number of species in function of their calculated range size (convex hull). (PNG 1422 kb) [file 12915_2017_356_MOESM5_ESM.png]

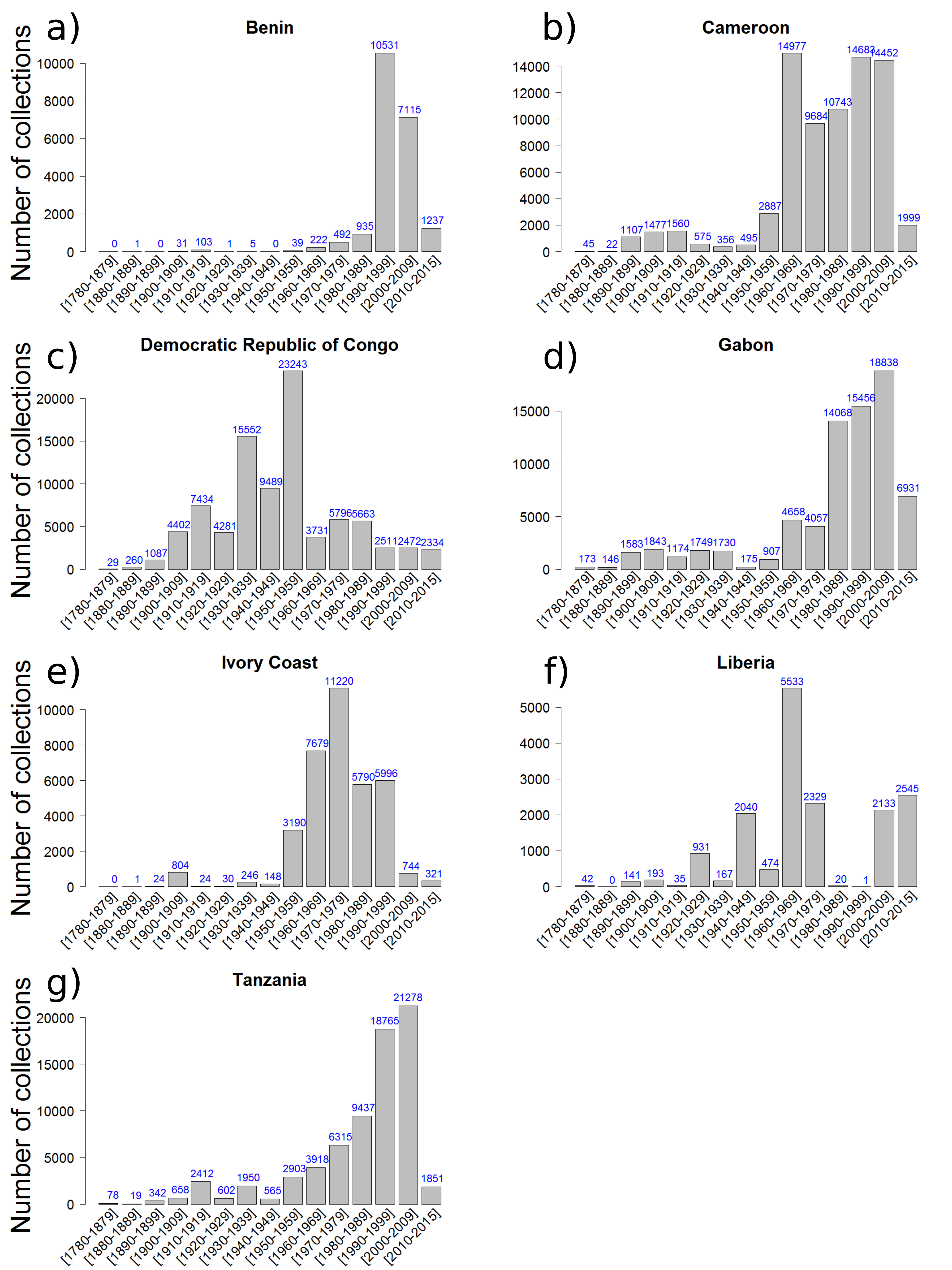

Supplement: Additional file 6: — Collecting history per country. Bar plots for a selection of tropical African countries showing the number of records collected in each period of time (5-year intervals). Plots based on herbarium records. (PNG 2657 kb) [file 12915_2017_356_MOESM6_ESM.png]

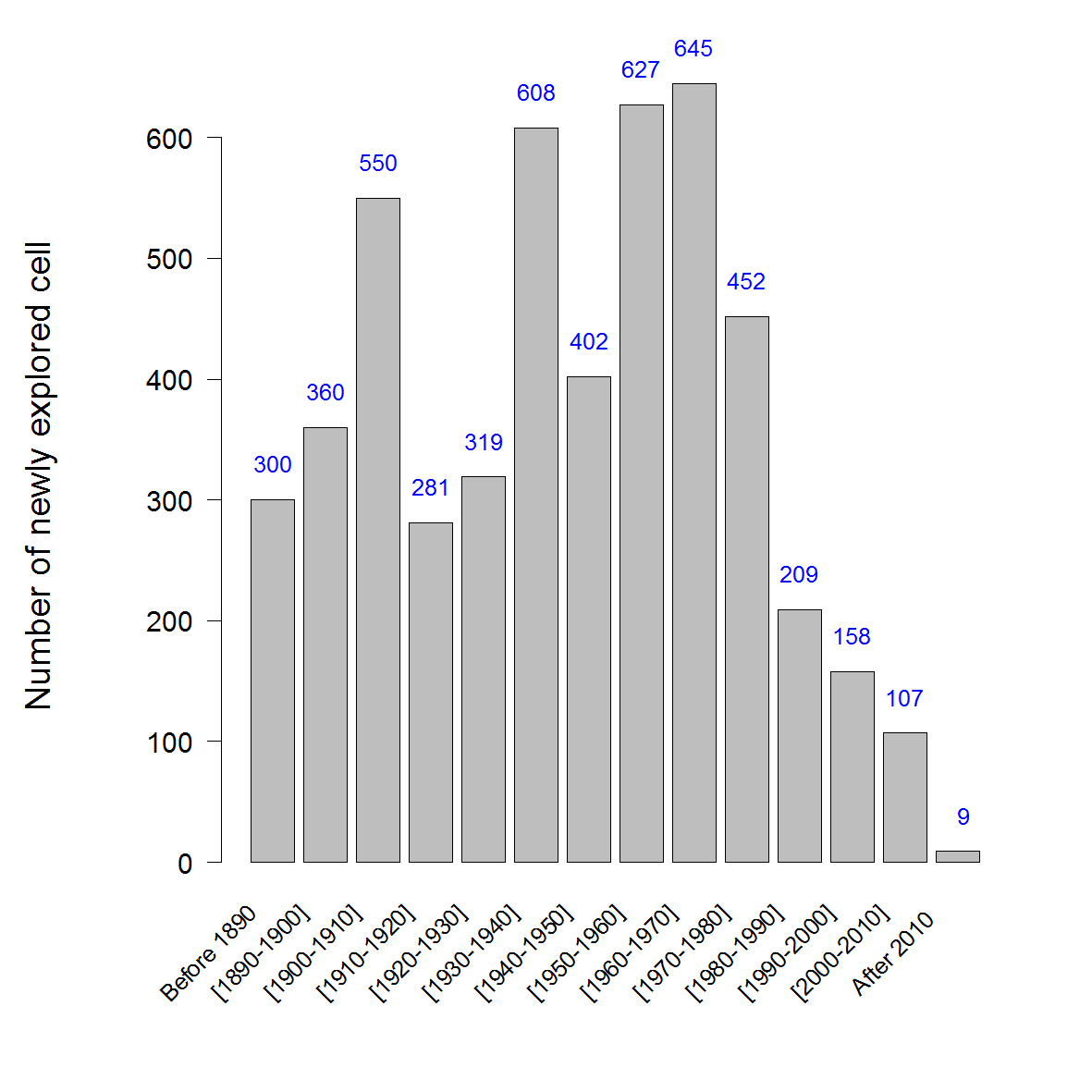

Supplement: Additional file 7: — Temporal exploration of tropical Africa per decade. The figure shows the newly explored 0.5° sampling units per decade. (PNG 21 kb) [file 12915_2017_356_MOESM7_ESM.png]

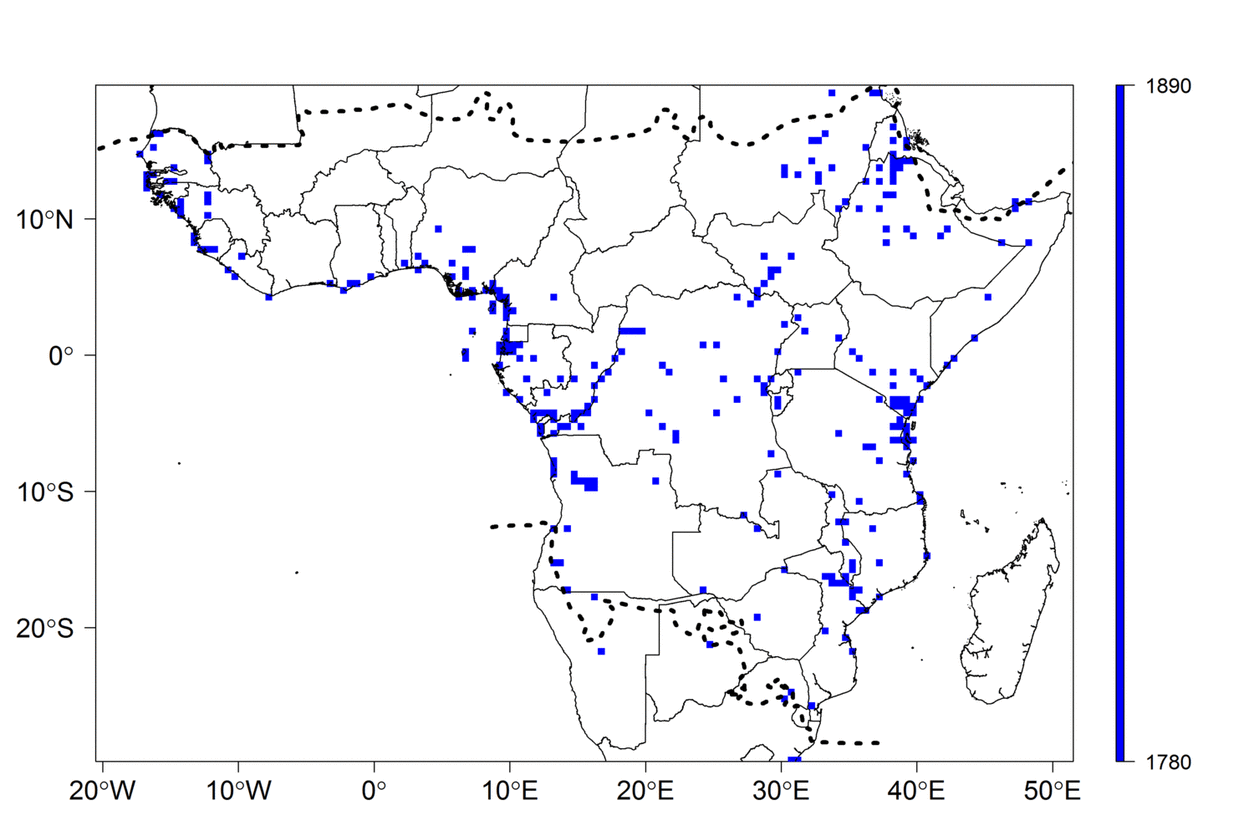

Supplement: Additional file 8: — A GIF animated figure of Fig. 5 by 10-year time slices. Also available at http://rainbio.cesab.org/. (GIF 2259 kb) [file 12915_2017_356_MOESM8_ESM.gif]
